# Functional characterization of iPSC-derived arterial- and venous-like endothelial cells

**DOI:** 10.1038/s41598-019-40417-9

**Published:** 2019-03-07

**Authors:** S. Rosa, C. Praça, P. R. Pitrez, P. José Gouveia, X. L. Aranguren, L. Ricotti, L. Silva Ferreira

**Affiliations:** 1 CNC UC- Center for Neurosciences and Cell Biology, University of Coimbra, 3004-517 Coimbra, Portugal; 2 IIIUC- Institute for Interdisciplinary Research, University of Coimbra, Casa Costa Alemão - Pólo II, Rua Dom Francisco de Lemos, 3030-789 Coimbra, Portugal; 3 The BioRobotics Institute, Scuola Superiore Sant’ Anna, Viale Rinaldo Piaggio 34, 56025 Pontedera, PI Italy; 4 Faculty of Medicine, University of Coimbra, 3000-354 Coimbra, Portugal; 5 Hematology and Cell Therapy Area, Clinica Universidad de Navarra, and Division of Oncology, Center for Applied Medical Research, University of Navarra, Pamplona, Spain

## Abstract

The current work reports the functional characterization of human induced pluripotent stem cells (iPSCs)- arterial and venous-like endothelial cells (ECs), derived in chemically defined conditions, either in monoculture or seeded in a scaffold with mechanical properties similar to blood vessels. iPSC-derived arterial- and venous-like endothelial cells were obtained in two steps: differentiation of iPSCs into endothelial precursor cells (CD31^pos^/KDR^pos^/VE-Cad^med^/EphB2^neg^/COUP-TF^neg^) followed by their differentiation into arterial and venous-like ECs using a high and low vascular endothelial growth factor (VEGF) concentration. Cells were characterized at gene, protein and functional levels. Functionally, both arterial and venous-like iPSC-derived ECs responded to vasoactive agonists such as thrombin and prostaglandin E2 (PGE_2_), similar to somatic ECs; however, arterial-like iPSC-derived ECs produced higher nitric oxide (NO) and elongation to shear stress than venous-like iPSC-derived ECs. Both cells adhered, proliferated and prevented platelet activation when seeded in poly(caprolactone) scaffolds. Interestingly, both iPSC-derived ECs cultured in monoculture or in a scaffold showed a different inflammatory profile than somatic ECs. Although both somatic and iPSC-derived ECs responded to tumor necrosis factor-α (TNF-α) by an increase in the expression of intercellular adhesion molecule 1 (ICAM-1), only somatic ECs showed an upregulation in the expression of E-selectin or vascular cell adhesion molecule 1 (VCAM-1).

## Introduction

Ischemic cardiovascular diseases (CVD) significantly impair quality of life being one of the leading causes of morbidity and mortality in industrialized countries^1^. Current treatments for CVD include pharmacological treatments and vascular surgeries; however, they show limited efficacy. As alternative, cell-based therapies are under investigation^2^. Cell-based therapies aim to replace dysfunctional vascular cells and promote the growth and repair of blood vessels. These therapies are also being investigated in the treatment of diabetic foot ulcers and peripheral vascular diseases, as these patients have microvascular complications and dysfunctional vascular cells^2^.

iPSCs are a relevant source of vascular cells since they have the ability to give rise to an unlimited number of ECs^2–6^. Patient-specific iPSC-derived ECs can constitute a source of autologous cells for several applications for the treatment of CVD, alone or in combination with a scaffold. iPSC-derived ECs are able to form functional blood vessels after transplantation on mouse animal models^4–7^ and zebrafish embryos^8^. ECs have now been differentiated using a variety of strategies and protocols (reviewed in^9^ and^10^). Some studies attempted to characterize but not to control the endothelial sub-phenotype^8,11^.

The specification of these ECs is of utmost importance for the use of these cells for regenerative medicine applications^9,10^. It is desirable that venous and arterial ECs will have different use according to the final application (e.g. grafts, patches or vascularization protocols) and tissue of engraftment. In the last 10 years, studies have documented the specification of ECs into venous and arterial ECs^6,12–17^. The first studies have differentiated endothelial progenitor cells into arterial and venous cells in the presence of non-defined media (i.e. containing serum). For example, iPSC-derived Flk1^+^ cells were differentiated into arterial and venous ECs by the modulation of cAMP and VEGF signaling pathways^18^. Flk1^+^ cells differentiated into venous and arterial cells after activation of VEGF or both VEGF and cAMP pathways, respectively. Others have differentiated iPSCs into arterial and venous ECs without the use of endothelial progenitor cells by manipulating the same signaling pathways^12^. More recently, studies have reported the generation of arterial and venous ECs from iPSCs in defined media^6,13,16^. In most cases, more than one signaling pathways have been manipulated to specify the sub-phenotype of ECs^13,16^. Only one study^6^ has documented the derivation of arterial and vein ECs by the control of a single signaling pathway (VEGF); however, no clear indication about the specification yield and stability of the derived cells was given. Despite the advances in the specification of iPSC-derived ECs, the functional properties of these sub-phenotypes have not been fully determined. Another issue not investigated is the functional properties of these iPSC-derived ECs after culture in scaffolds. Several scaffolds have been developed for the transplantation of iPSC-derived ECs^4,19,20^; however, in most cases, with mechanical properties different from native blood vessels (i.e. elastic modulus between 0.1 and 1 MPa^21,22^). Differences in mechanical compliance have been shown to increase the risk of thrombosis^23^ and intimal hyperplasia^24^.

Herein, we differentiated iPSC- derived EC precursor cells into arterial and venous-like ECs in chemically-defined conditions, modulating a single signaling pathway, i.e. VEGF. The derived cells were characterized at gene and protein levels. Our results showed that arterial-like ECs are closer to HUAECs while venous-like ECs closer to HUVECs. These cells were further characterized at functional level either in monoculture or seeded in scaffolds with elastic modulus values close to the native blood vessels. Our results indicated that both cells (i) responded to pro-inflammatory stimuli such as tumor necrosis factor-α (TNF-α), but at a lower extent than somatic ECs; (ii) responded to vasoactive agonists such as thrombin and prostaglandin E2 (PGE2), similarly to somatic ECs; (iii) responded differently to shear stress (arterial-like ECs more elongated than venous-like ECs); (iv) produced differently nitric oxide (arterial-like ECs more than venous-like ECs); (v) adhered, proliferated and formed a cell monolayer after seeding in scaffolds; and (vi) the cell monolayer did not induce platelet activation.

## Results

### Differentiation of hiPSCs into ECs in chemically-defined and serum-free conditions

hiPSCs were initially differentiated into mesoderm progenitors (Brachyury^pos^)^25^ followed by their differentiation into endothelial precursor cells (EPCs) that expressed residual levels of venous (e.g. COUP-TFII)^26^ and arterial (e.g. EphB2, receptor of ephrin B2)^27^ markers. To obtain mesoderm progenitors, undifferentiated cells were cultured with chemically defined medium (CDM; without serum)^28^ supplemented with 10 ng/ml BMP4 and 20 ng/ml FGF-β for 1.5 days and then 50 ng/ml BMP4 with 20 ng/ml FGF-β until day 5 (Supplementary Fig. S1). The mesoderm progenitors were further differentiated into EPCs by culturing the cells in media containing VEGF and Tβ4, known to be important for endothelial differentiation^3^ from day 5 until day 10. As control, iPSCs were also differentiated in CDM without supplements. The expression of mesoderm (*Brachyury*) and endothelial (*CD31*, *KDR* and *VE-cadherin*) mRNA transcripts during cell differentiation was evaluated by qRT-PCR analyses. The expression of *Brachyury* peaked at day 4 (Supplementary Fig. S1), *KDR* and *VE-cadherin* peaked at day 8, and the highest level of CD31 expression was observed at day 10 (Supplementary Fig. S1). In all cases the expression of mesoderm and EC markers was higher in cells cultured in CDM inductive media (CDMi) than CDM without supplements. To supplement the previous results, the expression of EC markers CD31 and KDR was analyzed by flow cytometry (Supplementary Fig. S1). At the beginning of the differentiation there was a residual expression of CD31 (1.0 ± 0. 2%) and low levels of KDR (10.6 ± 0.5%). The percentage of cells expressing both markers increased overtime (at least for 10 days) and was higher in cells differentiated in CDMi than in CDM without supplements. Cells at day 10 had an EC-like morphology and expressed CD31 at cell junctions as confirmed by immunostaining (Supplementary Fig. S2). Based on EC expression profile we isolated CD31^+^ cells as EPCs.

EPCs expressed general EC markers including CD31 and KDR; however, only 21.6 ± 2.4% of the cells were positive for the late EC marker, VE-cadherin, as quantified by flow cytometry (Supplementary Fig. S1). In addition, EPCs expressed residual levels of arterial marker EphB2 (2.0 ± 0.3%) and the venous marker COUP-TFII (2.4 ± 0.5%) as compared to HUAECs (EphB2: 35 ± 7.9%) and HUVECs (COUP-TFII: 92.5 ± 0.9%) (Supplementary Figs S1 and S2). Similarly, qRT-PCR data showed that EPCs had significantly lower levels of the venous markers *EphB4* than HUVECs and HUAECs (Supplementary Fig. S2). Overall, results suggest that our EPCs do not have yet a defined endothelial sub-phenotype.

### Derivation and characterization of arterial endothelial-like cells (AELCs)

**Figure 1:**
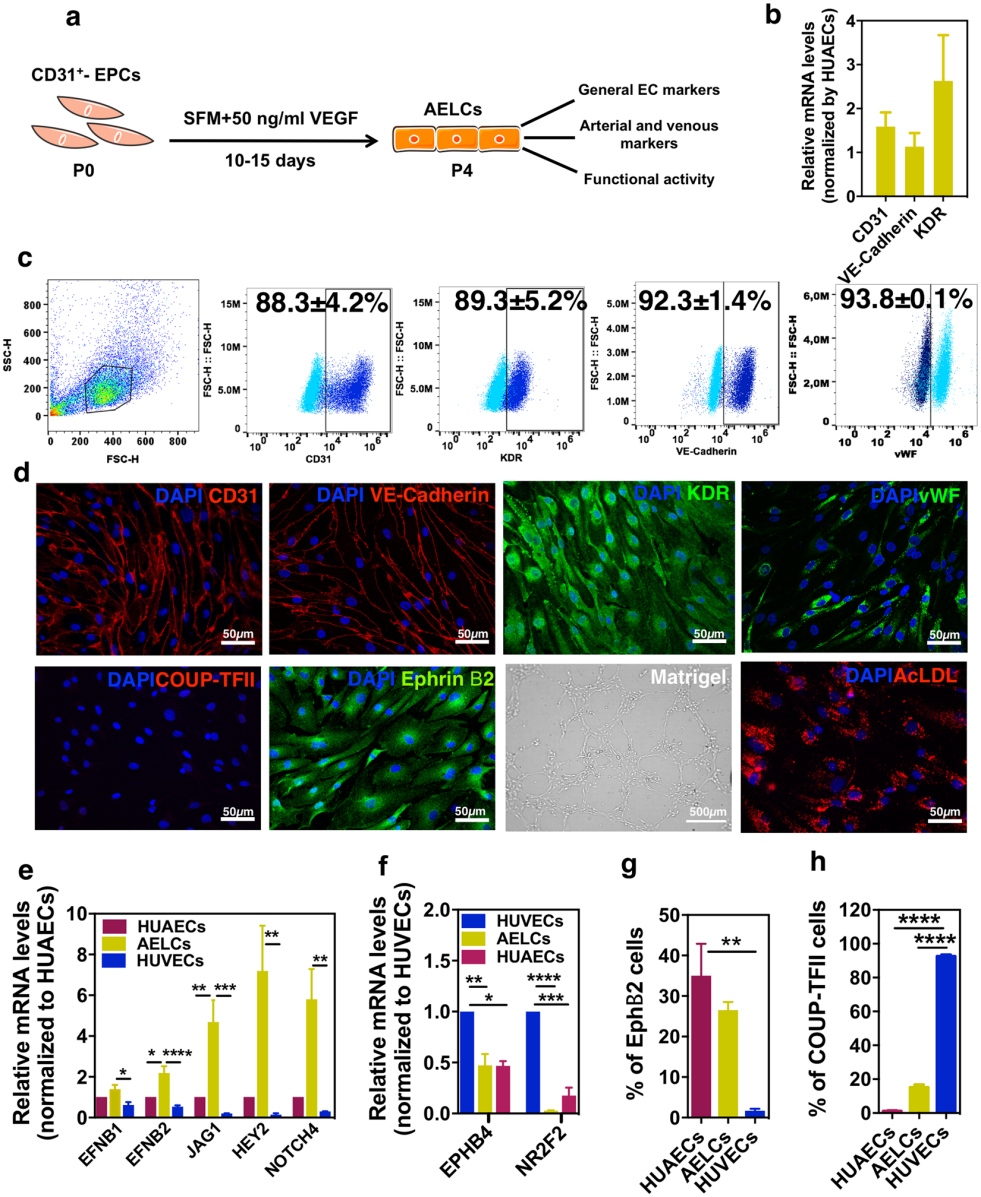
Derivation and characterization of AELCs. (**a**) Schematic representation of the protocol. EPCs were cultured in serum free medium supplemented with VEGF (50 ng/ml) for 10–15 days (4 passages). (**b**) qRT-PCR of general EC markers. Results were normalized by *GAPDH* and expressed relatively to HUAECs (n = 4). (**c**) FC analyses of EC markers (n = 3). (**d**) Expression and localization of general (CD31, KDR, VE-cadherin, vWF) as well as EC specification markers (COUP-TFII and Ephrin B2) by immunofluorescence. Functional activity of ECs as measured by their capacity to form cord-like structures in Matrigel and incorporate ac-LDL. (**e**) qRT-PCR of arterial markers (n = 4). Data was normalized by *GAPDH* and expressed relatively to HUAECs. (**f**) qRT-PCR of venous markers (n = 6). Data was normalized by *GAPDH* and expressed relatively to HUVECs. (**g**) Flow cytometry analyses of the arterial marker EphB2 (n = 3). (**h**) Quantification of the nuclei positive for COUP-TFII. Images were acquired in 6 different fields that contained an average of 300 cells. In (**e**,**f**,**g** and **h**) statistical analyses was performed by Kruskal-Wallis test followed by a Dunn’s multiple comparisons test. *p < 0.5, **p < 0.1, ***p < 0.001, ****p < 0.0001. Results are mean ± SEM.

EPCs were cultured in EC-serum-free medium supplemented with a high concentration of VEGF (50 ng/ml) for 4 passages (P0 to P4) (Fig. 1a). At gene level, iPSC-derived ECs (P4) expressed similar or higher mRNA levels of *CD31*, *VE-cadherin* and *KDR* than HUAECs (Fig. 1b). At protein level, the percentage of cells expressing CD31 and KDR was similar at P0 and P4 (CD31: 92.0 ± 3.3% to 88.3 ± 4.2%; KDR: 90.8 ± 2.5% to 89.3 ± 5.2%) while the percentage of cells expressing VE-cadherin increased significantly in cells from P0 to P4 (from 21.6 ± 2.4% at P0 to 92.3 ± 1.4% at P4) indicating a maturation process (Supplementary Fig. S1). This was further confirmed by a high percentage of cells expressing vWF (93.8 ± 0.1%) (Fig. 1c). Immunofluorescence results further confirmed that iPSC-derived ECs expressed CD31, KDR, VE-cadherin and vWF (Fig. 1d). At the functional level, these cells (P4) had the ability to uptake acetylated low-density-lipoprotein (Ac-LDL) (Fig. 1d and Supplementary Fig. S2) and form capillary like-networks on Matrigel (Fig. 1d and Supplementary Fig. S3).

Next, we evaluated the expression of arterial and venous markers in iPSC-derived ECs (P4). Increased expression of members of Notch canonical signaling pathway, including *NOTCH4*, *JAG1*, *HEY2*, *EFNB1*, *EFNB2*, *DLL4 and CXCR4* genes are associated with an arterial phenotype^10,29^. Conversely, increased expression of *NR2F2* and *EphB4* have been shown to be more restricted to the venous endothelium^26,30^. Gene expression analyses indicated that iPSC-derived ECs expressed higher levels of arterial markers than HUVECs or even HUAECs (Fig. 1e and Supplementary Fig. S4). In addition, cells expressed less *NR2F2* and *EphB4* than HUVECs (Fig. 1f). These results have been confirmed at protein level. Approximately 27% and 100% of iPSC-derived ECs (P4) expressed EphB2 (receptor of ephrin B2) and EphrB2 (ephrin B2 ligand), respectively, near the cell percentages observed in HUAECs (Fig. 1g, Supplementary Fig. S2). As expected, a very low percentage of iPSC-derived ECs (P4) expressed the venous marker COUP-TFII (ca. 15%) (Fig. 1h and Supplementary Fig. S2). Importantly, the arterial phenotype in iPSC-derived ECs was maintained for at least 9 passages, as evaluated by the percentage of EphB2 positive cells (Supplementary Fig. S4). To confirm that the differentiation protocol was extensive to other human pluripotent stem cells, human embryonic stem cells (hESCs) were used. Similar to iPSCs, increased expression of arterial genes was obtained in hESC-derived ECs (Supplementary Fig. S5). Taken together, based in the general expression of EC markers, expression of arterial markers and EC functional activity, the cells were named as AELCs. Our results show that EPCs cultured in EC-serum-free medium supplemented with a high concentration of VEGF gave rise to AELCs. From 1 million undifferentiated iPSCs we obtained approximately 7.5 million of AELCs after 20 days of differentiation.

### Derivation and characterization of venous endothelial-like cells (VELCs)

**Figure 2:**
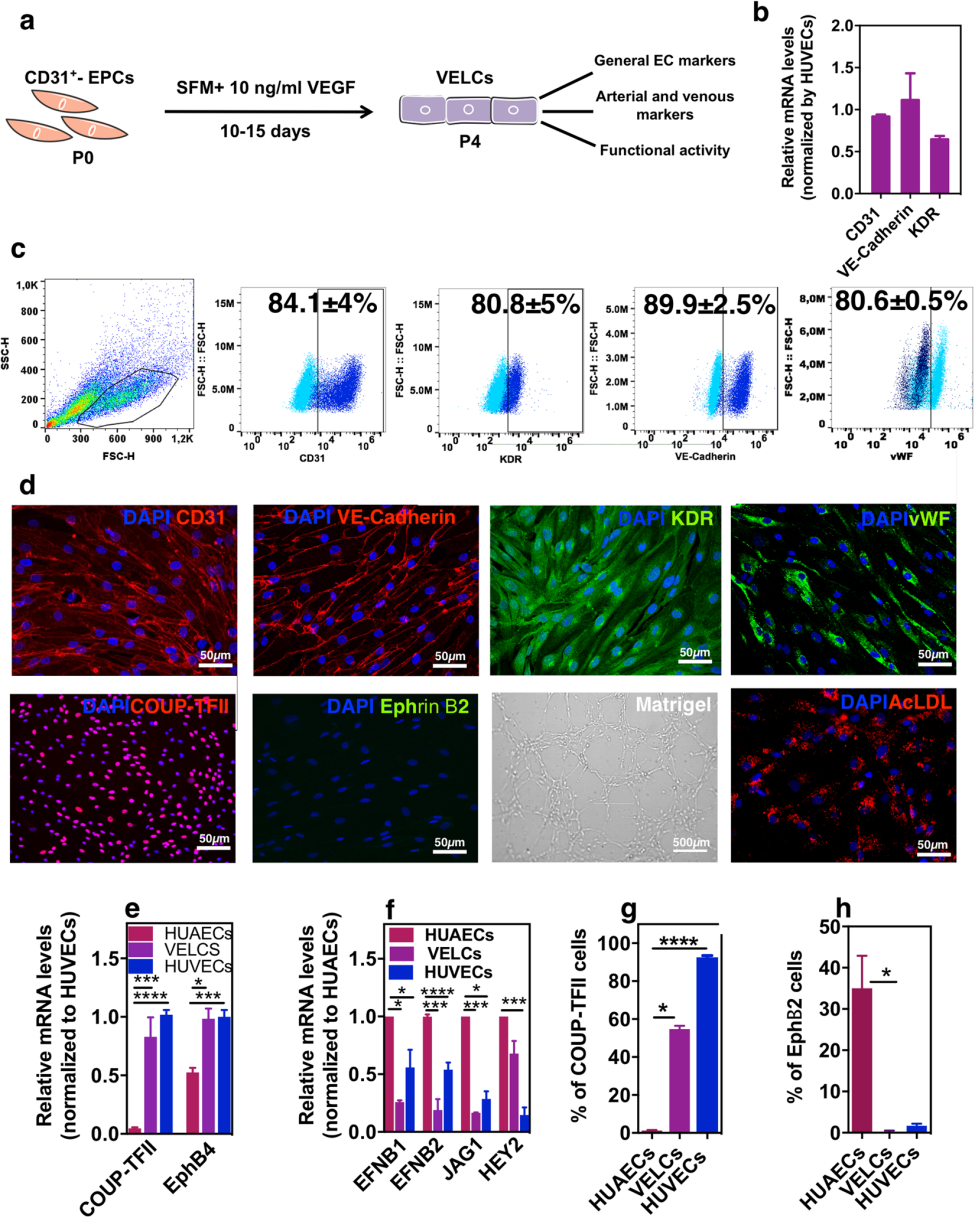
Derivation and characterization of VELCs. (**a**) Schematic representation of the protocol. The EPCs were cultured in serum free medium supplemented with VEGF (10 ng/ml) for 10–15 days (4 passages). (**b**) qRT-PCR of general EC markers. Data were normalized by *GAPDH* and expressed relatively to HUVECs (n = 4). (**c**) FC analyses of EC markers (n = 3). (**d**) Expression and localization of general (CD31, KDR, VE-cadherin, vWF) and specification EC markers (COUP-TFII and Ephrin B2) by immunofluorescence. Functional activity of ECs measured by their capacity to form cord-like structures in Matrigel and incorporate ac-LDL. (**e**) qRT-PCR for venous markers. Data was normalized by *GAPDH* and expressed relatively to HUVECs (n = 3). (**f**) qRT-PCR of arterial markers. Data were normalized by *GAPDH* and expressed relatively to HUAECs (n = 3). (**g**) Quantification of COUP-TFII. Images were acquired in 6 different fields that contained an average of 300 cells. (**h**) Flow cytometry analyses of the arterial marker EphB2 in ECs (n = 3). In (**e**–**h**) statistical analyses were performed by Kruskal-Wallis test followed by a Dunn’s multiple comparisons test. *p < 0.5, **p < 0.1, ***p < 0.001, ****p < 0.0001. Results are mean ± SEM.

EPCs were cultured in EC-serum-free medium supplemented with a low concentration of VEGF (10 ng/ml) for 4 passages (P0 to P4) (Fig. 2a). At gene level, iPSC-derived ECs (P4) expressed similar mRNA levels of *CD31*, *KDR* and *VE-cadherin* than HUVECs (Fig. 2b). At protein level, the expression of CD31 and KDR slightly decreased from P0 to P4 (CD31: 92.0 ± 3.3% to 84.1 ± 4%; KDR: 90.8 ± 2.5% to 80.8 ± 5%) while the expression of VE-cadherin increased significantly (from 21.6 ± 2.4% at P0 to 89.9 ± 2.5% at P4). At P4, approximately 81% of the cells were positive for vWF, indicating an EC maturation process (Fig. 2c). Immunofluorescence results further confirmed that iPSC-derived ECs expressed CD31, KDR, VE-cadherin and vWF (Fig. 2d). At the functional level, iPSC-derived ECs (P4) had the capacity to uptake Ac-LDL (Fig. 2d and Supplementary Fig. S2) and to form a capillary like-network on top of Matrigel (Fig. 2d and Supplementary Fig. S3).

Next, we evaluated the expression of arterial and venous markers in iPSC-derived ECs. These cells (P4) showed high mRNA expression of the venous marker *NR2F2* and *EphB4* (Fig. 2e) and low mRNA expression of the arterial markers *HEY2*, *EFNB1*, *EFNB2*, *JAG1 DLL4 and CXCR4* as compared to HUAECs (Fig. 2f and Supplementary Fig. S4). At protein level, the percentage of iPSC-derived ECs (P4) expressing COUP-TFII and EphB2/Ephrin B2 was ca. 55% and 0% (Fig. 2d,g,h and Supplementary Fig. S2). The percentage of COUP-TFII positive cells was significantly higher than the one found for EPCs (1.1 ± 0.3%) and AELCs (15 ± 1.2%) but lower than the one found for HUVECs (92.5 ± 0.9%). The venous specification of iPSC-derived ECs was maintained for at least 9 passages as evaluated by the percentage of COUP-TFII positive cells that was similar to P4 cells (Supplementary Fig. S5). To confirm that the differentiation protocol was extensive to other human pluripotent stem cells, hESCs were used. Similar to iPSCs, increased expression of venous genes was obtained in hESC-derived ECs (Supplementary Fig. S6). Taken together, based on the general expression of EC markers, expression of venous markers and EC functional activity, the cells were named as VELCs. Our results show that EPCs cultured in EC-serum-free medium supplemented with a low concentration of VEGF gave rise to VELCs. From 1 million undifferentiated iPSCs we obtained approximately 7.5 million of VELCs after 20 days of differentiation.

### Functional characterization of AELCs and VELCs in monoculture

**Figure 3:**
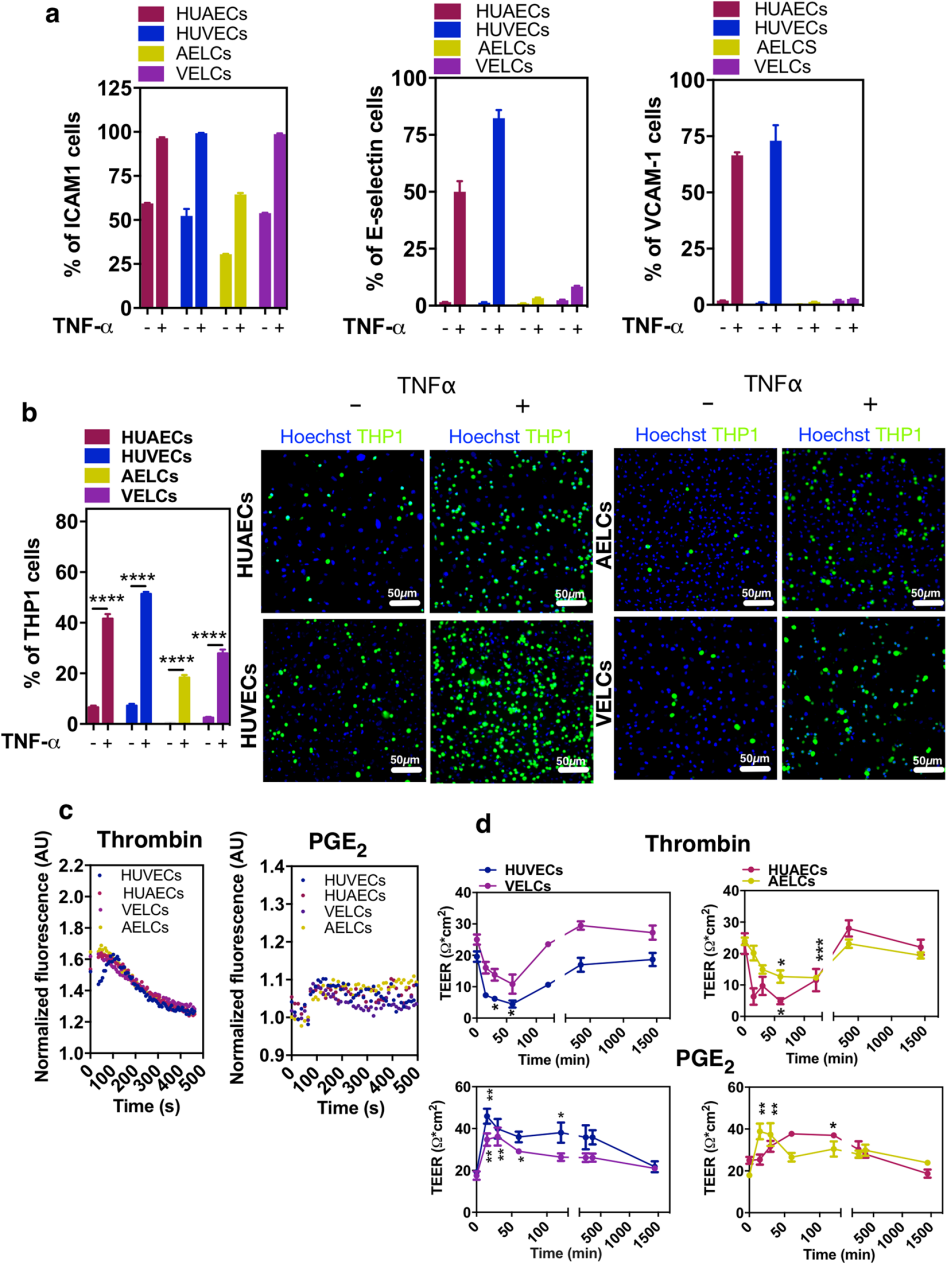
Functional characterization of AELCs and VELCs. (**a**) Flow cytometry analyses of adhesion molecules in ECs before and after exposure to TNF-α (10 ng/ml) for 24 h (n = 3). (**b**) EC monolayers treated or not with TNF-α (10 ng/ml) for 6 h were co-cultured for 30 min with CSFE labeled THP1. Number of adherent THP1 cells was counted and expressed as percentage relative to the total number of nuclei (ECs and THP1). Representative images for the adhesion of THP1 cells are shown (n = 3). (**c**) Variation of intracellular Ca^2+^ in FURA-2-loaded ECs in response to thrombin and PGE_2_. Traces are representative of 6 replicas for each condition. (**d**) Changes in TEER of cells treated with thrombin or PGE_2_ (n = 3). In (**b**) statistical analyses were performed by a Mann-Whitney test. In (**d**) statistical analyses were performed by a Kruskal Wallis test followed by a Dunn’s multiple comparisons test. Statistical significance in each time point is relatively to the initial time point (time 0). *p < 0.5, **p < 0.1, ***p < 0.001, p**** < 0.0001. Results are mean ± SEM.

We asked whether AELCs or VELCs could respond to pro-inflammatory stimuli such as TNF-α by assessing the expression of adhesion molecules such as E-selectin, ICAM-1 and VCAM-1. The expression of these adhesion molecules is critical for the adhesion of monocytes to ECs^31^. Cells were treated with TNF-α (10 ng/ml) for 24 h and the expression of adhesion molecules monitored by flow cytometry. Both AELCs and VELCs showed an up-regulation in the expression of ICAM-1 as observed for HUAECs and HUVECs (Fig. 3a). The mean fluorescence intensity (MFI), as assessed by flow cytometry, was higher in VELCs than in AELCs, similarly to what was observed in HUVECs *versus* HUAECs. Similar trend was observed at gene level (Supplementary Fig. S7). Interestingly, a relatively low percentage of AELCs and VELCs expressed E-Selectin and VCAM-1 in response to TNF- α in comparison with HUAECs and HUVECs, suggesting a certain degree of immaturity in our iPSC-derived ECs (Fig. 3a).

Since ECs were activated by pro-inflammatory cytokines we complemented the previous results with a monocyte-EC adhesion assay to determine whether adhesion molecules were functional in mediating monocyte adhesion to ECs and to determine whether this activity was different between arterial and venous ECs^31,32^. For that purpose, cells were treated with TNF-α (10 ng/ml) for 6 h, washed, co-cultured with monocytes fluorescently labeled for 30 min and the unbound monocytes removed by washing. Our results showed that the adhesion of THP1 cells to TNF-α-treated AELCs or VELCs was higher than non-treated ECs (Fig. 3b). Yet, the percentage of adherent THP1 cells in TNF-α-treated AELCs or VELCs was lower than the one observed in TNF-α-treated HUVECs or HUAECs. It is likely that the lower adhesion of THP1 in TNF-α-treated AELCs and VELCs was related to the lower expression of E-Selectin and VCAM-1 as the one observed in TNF-α-treated HUAECs and HUVECs.

A general property of ECs is their ability to respond to vasoactive agents and to rearrange junctional and cytoskeletal proteins in response to physiological stimulus to maintain a tight dynamic barrier^33^. Indeed, AELCs and VELCs responded to the vasoactive agonists such as thrombin and PGE_2_, similar to HUVECs or HUAECs, by increasing the intracellular levels of Ca^2+^ (Fig. 3c). As a consequence, both AELC and VELC monolayers showed a decrease or increase in basal trans-endothelial electrical resistance (TEER) levels in case they were stimulated with thrombin or PGE_2_, respectively (Fig. 3d).

**Figure 4:**
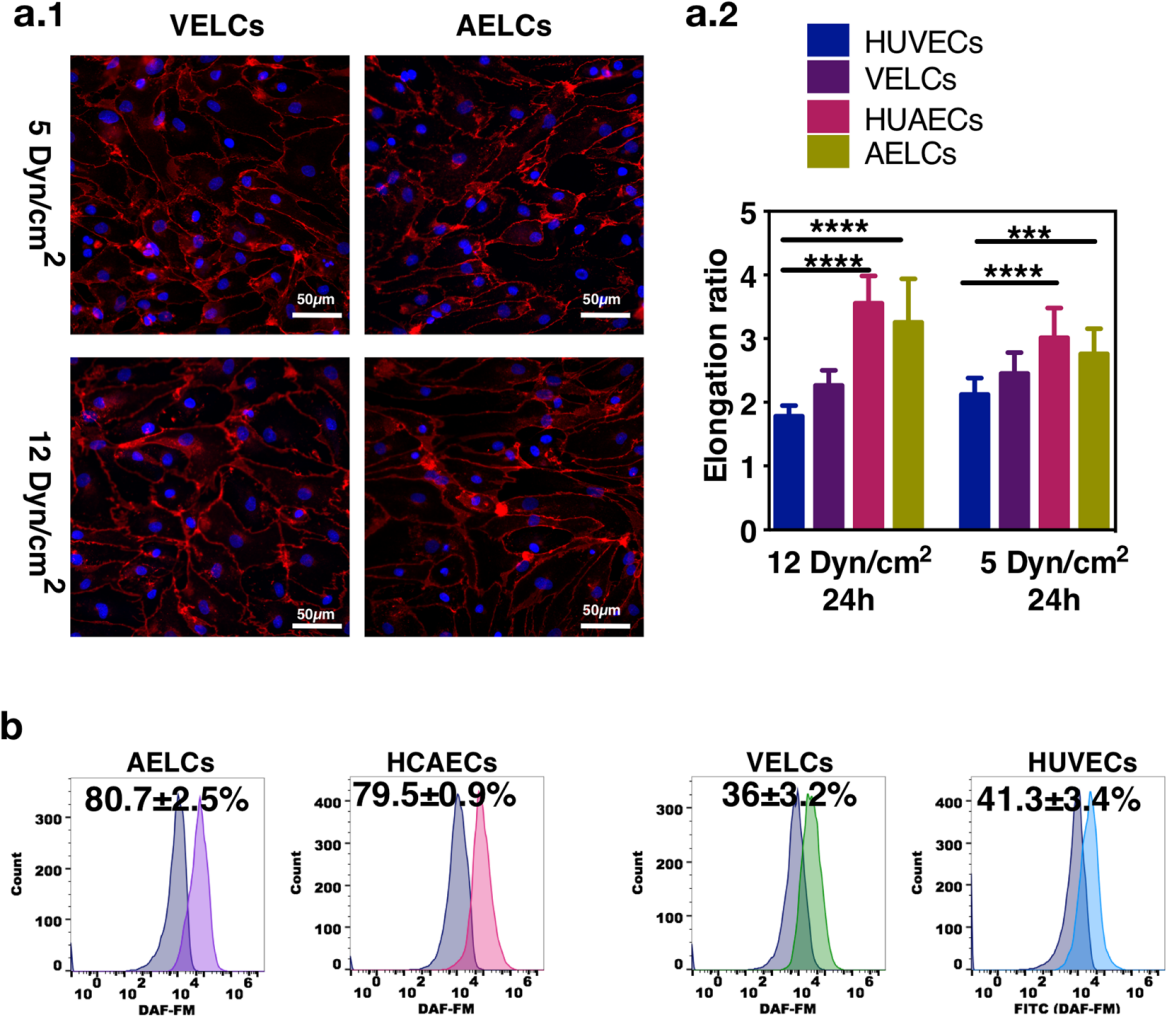
Functional characterization of iPSCs-derived AELCs and VELCs. (**a**.**1**) Representative images of AELCs or VELCs cultured under flow shear stress values for 24 h. Cells were stained for VE-cadherin. (**a**.**2**) Elongation ratio (calculated by the ratio of cell length to width) of AELCs, VELCs, HUAECs and HUVECs cultured under different flow shear stress values for 24 h. Quantifications were done in 3 microfluidic systems per experimental condition (approximately 150 cells were counted per each microfluidic system). Statistical analyses were performed using a Kruskal-Wallis test, followed by a Dunn’s multiple comparisons test. (**b**) Nitric Oxide (NO) production on AELCs, VELCs, HUVECs and HCAECs was measured using the probe DAF-FM. DAF-FM is non-fluorescent until it reacts with NO forming benzotriazole, a fluorescent compound. Fluorescence intensity was analyzed by flow cytometry. In (**a**–**c**) results are mean ± SEM, n = 3. *p < 0.5, **p < 0.1, ***p < 0.001, p**** < 0.0001.

Arterial and venous ECs show different activities regarding, NO production and elongation to shear stress^13^. Our results showed that AELCs and HUAECs had higher elongation, particularly to high shear stress (e.g. 12 dyn/cm^2^), than VELCs or HUVECs (Fig. 4a.1a.2). Moreover, the NO production was higher on AELCs than VELCs, and thus explaining the fact that AELCs had lower monocyte adhesion than VELCs (NO prevents monocyte adhesion) (Fig. 4c).

Overall, our results show that AELCs and VELCs respond to vasoactive agents such as thrombin and PGE_2_ as somatic ECs including HUVECs and HUAECs; however, they show a lower pro-inflammatory response to TNF-α than somatic HUVECs or HUAECs. Our results further demonstrate that AELCs produced higher NO and elongation to shear stress than VELCs.

### Functional characterization of AELCs and VELCs in a scaffold with mechanical properties similar to blood vessels

The scaffolds prepared in this work (screened from different polymers and concentrations) were based on stable ultrathin polymeric nanofilms. Nanofilms were prepared from degradable polymers, including poly(L-lactic acid) (PLLA) and poly(caprolactone) (PCL), and non-degradable polymers such as poly(dimethylsiloxane) PDMS (Supplementary Fig. S8). PCL was chosen because *in vivo* studies have demonstrated that PCL scaffolds are suitable to engineer blood vessels^34–36^. PDMS and PLLA have also been used in several biomedical applications and they allow EC adhesion and proliferation^37,38^. Nanofilms were prepared with an area of 2 × 2.5 cm^2^ and with a thickness between 40 and 60 nm (Supplementary Fig. S8) as measured by atomic force microscopy (AFM). As evaluated by traction tests, the nanofilm elastic modulus ranged between 0.98 ± 0.4 and 16.5 ± 3.1 MPa for PCL, 0.2 ± 0.02 and 0.9 ± 0.06 MPa for PDMS and 1.4 ± 0.3 to 31.2 ± 8.9 MPa for PLLA, respectively (Supplementary Fig. S8). Based on the mechanical properties we selected PCL (10 mg/mL) and PDMS (1/10) nanofilms for further analyses because the elastic modulus was in the range observed in arteries, and because the PLLA nanofilms with low elastic modulus were very fragile and often crashed during manipulation. As measured by AFM, the average surface roughness (Ra) of PCL and PDMS nanofilms was 0.007 ± 0.0004 μm and 0.01 ± 0.002 μm, respectively (Supplementary Fig. S8).

**Figure 5:**
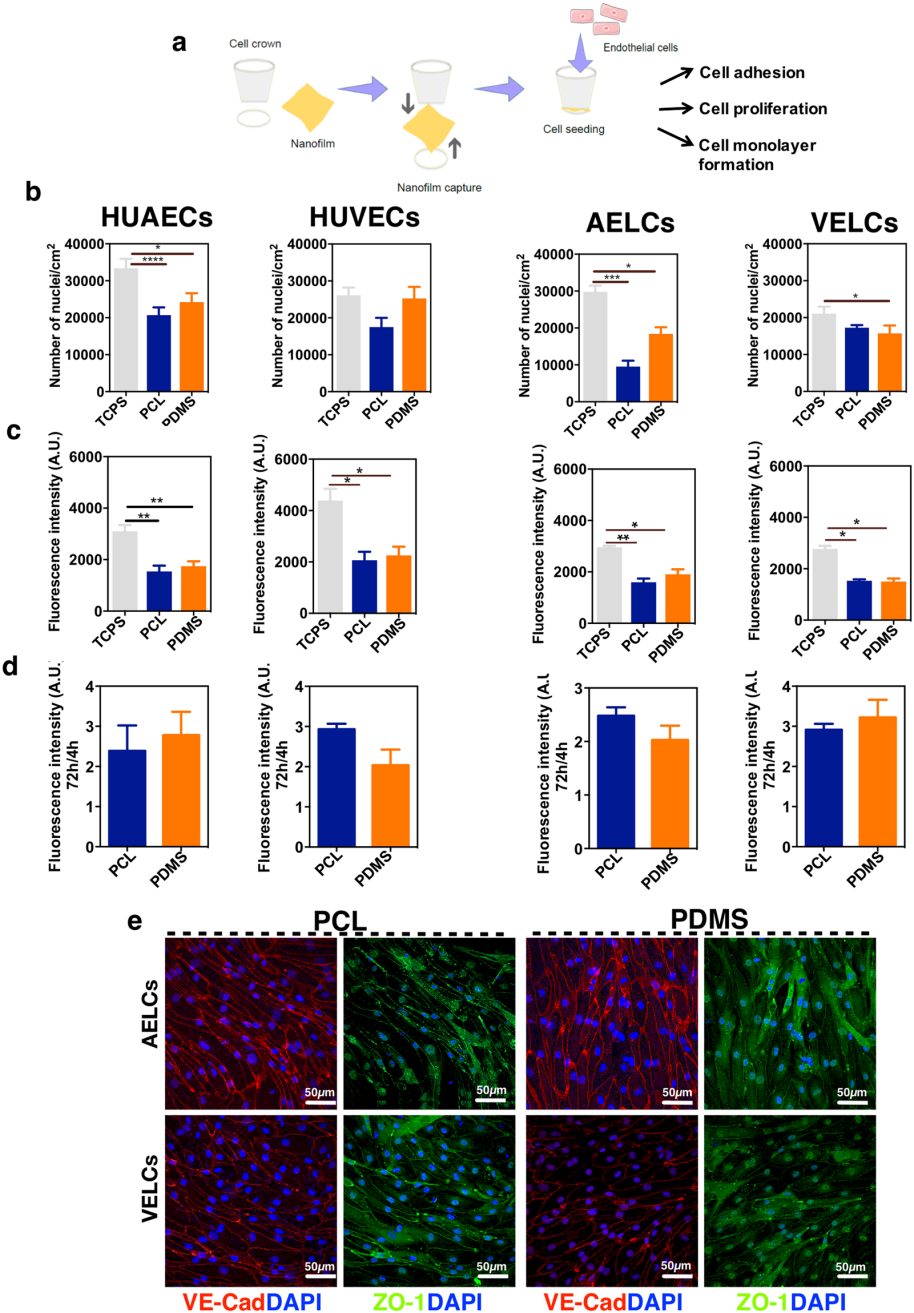
Characterization of EC- nanofilm interactions. (**a**) Schematic representation of the assembly of the nanofilms onto the cell crown before cell seeding. (**b**) Cell adhesion to the different substrates. Cell number per substrate was quantified at 4 h after cell seeding. Images were taken from 6 random fields by condition. Each condition was performed in triplicate (n = 3). (**c**) Cell viability. Cell viability was assessed at time 4 h after seeding, using Presto Blue assay. Each condition was performed in triplicate (n = 3). (**d**) Cell proliferation. Cell proliferation in each substrate was calculated by dividing the fluorescence intensity at 72 h by the one at 4 h. (**e**) Expression and localization of EC markers in AELCs and VELCs cultured on top of nanofilms for 3 days. In (**b**–**d**) statistical analyses were performed by Kruskal Wallis test followed by a Dunn’s multiple comparisons test. *p < 0.5, **p < 0.1, ***p < 0.001, p**** < 0.0001. Results are mean ± SEM.

Nanofilms were mounted in a cell culture insert that kept the nanofilms immobilized during cell culture (Fig. 5a). Cell adhesion in PDMS nanofilms and PCL nanofilms was quantified 4 h after cell seeding by quantifying the number of Hoechst positive nuclei. Our results showed that approximately 60–70% of the cells adhered to PCL and PDMS nanofilms, relatively to TCPS condition (Fig. 5b). These cells were viable as assessed by a Presto Blue assay (Fig. 5c). Cell proliferation results, obtained by the ratio of cell viability (Presto Blue assay) at 72 h and 4 h, showed that all cell types proliferated similarly in both PCL and PDMS nanofilms (Fig. 5d). The iPSC-derived AELCs and VELCs cultured for 72 h on top of the nanofilms were able to form a monolayer and they expressed VE-cadherin and ZO-1 (Fig. 5e). We found a significant higher VE-cadherin intensity for both VELCs and AELCs cultured for 3 days in PCL nanofilms in comparison with VELCs and AELCs cultured in PDMS nanofilms (Supplementary Fig. S9).

**Figure 6:**
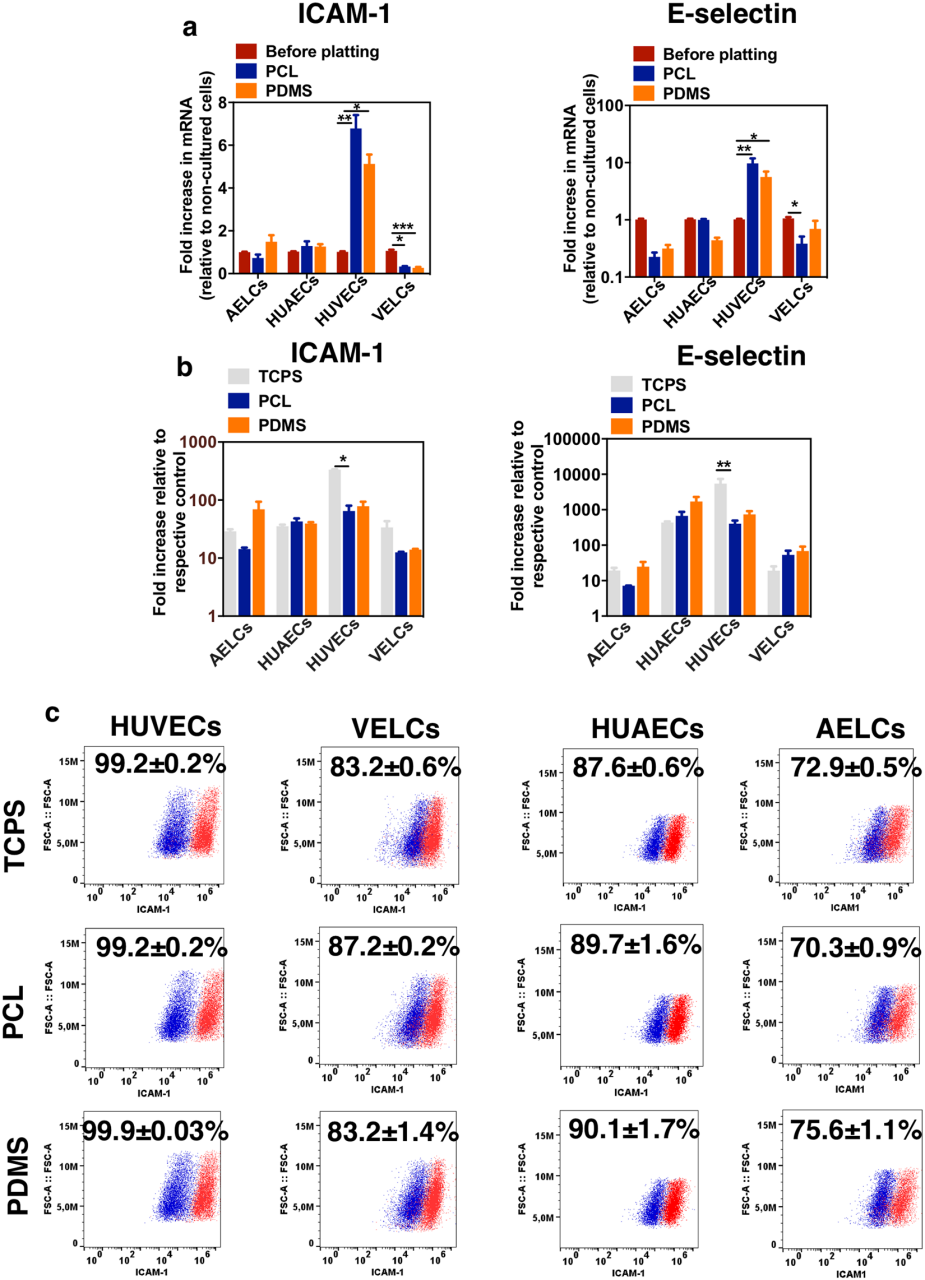
Inflammatory profile of ECs cultured in nanofilms. (**a**) mRNA levels of *ICAM-1* and *E-Selectin* in ECs cultured on different substrates for 72 h. Expression of *ICAM-1* and *E-Selectin* was normalized by *GAPDH* and expressed relatively to the levels before platting (n = 6). (**b**) mRNA levels of *ICAM-1* and *E-Selectin* in ECs cultured on different substrates for 72 h and then activated with TNF-α (10 ng/ml) for 6 h, as evaluated by qRT-PCR. Results are expressed as fold increase relative to non-treated cells (n = 3). (**c**) Flow cytometry analyses of ICAM-1 expression in ECs before (dark blue) and after (red) treatment with TNF-α (10 ng/ml) for 24 h. Percentages shown were calculated based on the basal levels of ICAM-1 (n = 3). In (**a**,**b**) statistical analyses were performed by Kruskal-Wallis test followed by a Dunn’s multiple comparisons  test. *p < 0.5, **p < 0.1, ***p < 0.001, p**** < 0.0001. Results are mean ± SEM.

Since scaffold modulus can alter the immunogenicity of ECs^39^, we evaluated the expression of *ICAM-1*, *E-selectin and VCAM-1* in ECs cultured on top of the nanofilms. *VCAM-1* mRNA levels were below the detection limits for all the cells analyzed (data not shown). *ICAM-1* and *E-selectin* mRNA levels were altered in HUVECs after culture on PDMS and PCL nanofilms (up-regulation) and VELCs after culture in PCL and PDMS nanofilms (down-regulation) (Fig. 6a).

Next, we evaluated whether the inflammatory response of cells cultured on both nanofilms was affected after exposure to TNF-α for 6 h. *ICAM1* and *E-selectin* mRNA levels in TNF-α- activated cells were assessed by qRT-PCR and normalized by the ones in non-treated cells. A significant up-regulation of *ICAM-1* and *E-selectin* mRNA levels was observed for all conditions. However, the up-regulation of *E-selectin* mRNA levels in TNF-α-treated AELCs or VELCs were lower than the ones observed in HUVECs or HUAECs (Fig. 6b). These results are in line with the results in Fig. 3a.

To further confirm the inflammatory response profile of cells cultured on nanofilms we quantified the expression of ICAM-1 and E-selectin by flow cytometry. Cells were cultured in PCL or PDMS nanofilms and tissue cultured polystyrene (as control) and then treated with TNF-α for 24 h. Flow cytometry results show that HUVECs, AELCs and VELCs cultured on the nanofilms expressed similar basal levels of ICAM-1 protein as the ones cultured in TCPS (Fig. 6c and Supplementary Fig. S9). Therefore, the increased ICAM-1 mRNA levels observed in HUVECs cultured on PCL and PDMS nanofilms (Fig. 6a) were not correlated with a high ICAM-1 protein expression, at least after 24 h. TNF-α activated HUVECs, VELCs, HUAECs and AELCs express similar ICAM-1 levels regardless of the substrate (Fig. 6c). Regarding E-selectin, TNF-α-activated AELCs and VELCs cultured in the nanofilms or TCPS expressed negligible (below 4%) values of E-selectin (Supplementary Fig. S10). In contrast, TNF-α-activated HUVECs or HUAECs cultured in the nanofilms or TCPS expressed high values of E-selectin (approximately 50 to 60% of the cells). Overall, our results show that cell culture onto nanofilms did not change significantly basal ICAM-1 and E-selectin expression. In addition, we found that cells respond to TNF-α regardless of the substrate in which they were cultured; however, with different magnitude. The results indicate that iPSC-derived ECs have a lower inflammatory profile in response to TNF-α than HUVECs or HUAECs.

**Figure 7:**
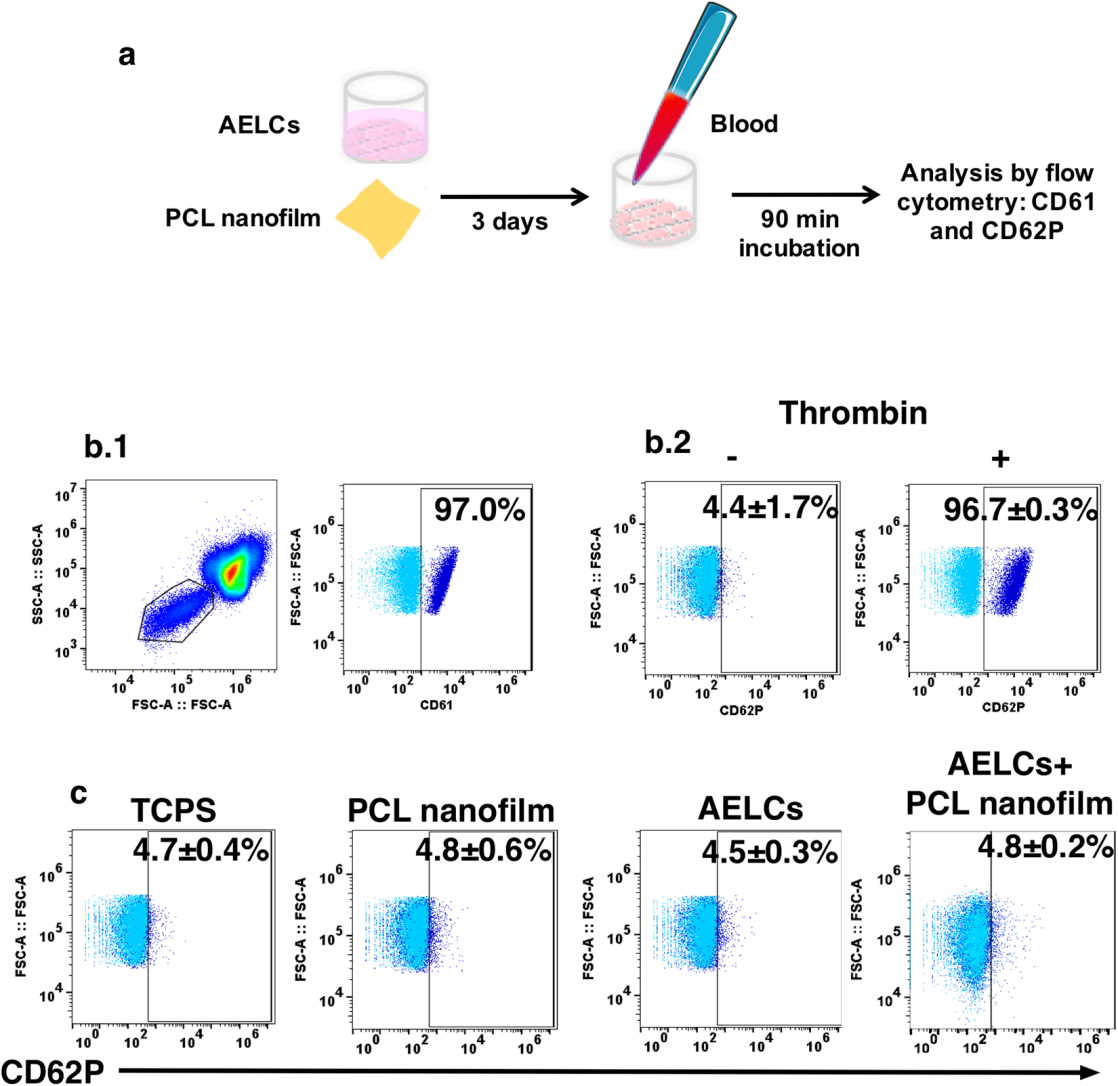
Hemocompatibility of nanofilms seeded with iPSCs-derived ECs. (**a**) AELCs were maintained in culture for 3 days. PCL nanofilms were maintained in the same conditions. Human blood was incubated for 90 min at 37 °C with the cells and/or nanofilms, and TCPS. (**b**) Flow cytometry analysis of blood after incubation. (**b**.**1**) FSC:SSC plot showing the gating of platelets. (**b**.**2**) Analysis of CD62P in human blood platelet population before and after incubation with thrombin. (**c**) Analyses of CD62P positive events in human blood platelet population after incubation with TCPS, AELCs and PCL nanofilms with or without AELCs. Results are mean ± SEM (n = 3).

The hemocompatibility of the nanofilms seeded with iPSC-derived ECs was evaluated by a platelet activation assay. For these tests, we have used PCL nanofilms, AELCs, and PCL nanofilms seeded with AELCs. We have chosen PCL rather than PDMS nanofilms for these analyses because PCL nanofilms had a better performance in maintaining the EC phenotype (Supplementary Fig. S9) and they are degradable. iPSC-derived ECs were maintained in culture for 3 days while PCL nanofilms were immersed in culture medium for the same period of time (Fig. 7a). Platelet activation was measured by the flow cytometric quantification of CD62P (P-selectin) expression^40^. CD62P is a component of the α-granule membrane of resting platelets that translocates to the surface in consequence of platelet activation. Tyrode’s buffer diluted blood was incubated for 1 h with the cells and/or surfaces, afterwards blood was stained for CD61 to identify platelets and CD62P to identify activated platelets^40^.

As expected, thrombin induced a strong platelet activation (Fig. 7b.2). PCL nanofilms had no significant effect in platelet activation, as the percentage of CD62P^+^ platelets (4.8 ± 0.6%) was similar to TCPS (4.7 ± 0.4%) (Fig. 7c). AELCs alone did not activate platelets. Furthermore, AELCs in culture on PCL nanofilms did not induce (4.8 ± 0.2%) platelet activation.

## Discussion

This study describes a protocol to derive arterial or venous-like ECs in chemically defined conditions by the modulation of VEGF signaling pathway and reports EC functional properties either in monoculture or after culture in a scaffold with mechanical properties similar to blood vessels.

Although recent studies have documented the differentiation of iPSCs into arterial/venous ECs^6,12,13,16^; only some protocols were performed in serum-free conditions^6,13,16^. In this study, the differentiation of iPSCs into arterial/venous ECs was performed in two steps in chemically defined medium. The protocol is based on the use of BMP4 and FGF-β to induce mesoderm formation followed by VEGF and Tβ4 to drive the differentiation into endothelial precursor cells (CD31^pos^/KDR^pos^/VE-cad^med^/EphB2^neg^/COUP-TF^neg^). The obtained EPCs were then differentiated into arterial or venous-like ECs by the use of a high and low concentration of VEGF, respectively. During the submission of this work, two studies have demonstrated the derivation of arterial and venous ECs in serum-free culture medium from iPSCs^13,16^. In both studies, VEGF pathway among other signaling pathways including AMPc, WNT, FGF2, insulin, BMP4 and PDGF-BB have been regulated for arteriovenous specification. Our protocol has the advantage of simplicity by the regulation of a single signaling pathway to obtain AELCs and VELCs. For 1 million undifferentiated iPSCs we could obtain 7.5 million of AELCs or VELCs after 20 days of differentiation, while previous protocols obtained 1.7 million AELCs after 6 days^13^ or 4 million AELCs after 10 days^16^ from the same number of undifferentiated iPSCs. Moreover, the cells obtained in this work (arterial and venous-like ECs) were characterized functionally at a level that was not demonstrated in previous studies. Such characterization is relevant for their final biomedical application.

VEGF signaling has been used to modulate the specification of ECs, under serum-free conditions. Previous studies have used a combination of factors (VEGF, insulin and BMP)^13^, AMPc^16^ or VEGF^6^ for the arteriovenous specification. Although the modulation of VEGF signaling has been used in a previous study, it was unclear the number of ECs obtained at the end of the protocol^6^. VEGF signaling modulates EC specification by acting upstream of Notch signaling, a gateway for EC specification^32^.

Arterial and venous ECs have different properties. Venous endothelium is more adhesive for leukocytes than arterial endothelium^31^. We found a trend for higher adhesion of THP1 monocyte cells in VELCs than in AELCs and HUVECs than HUAECs treated with TNF-α, suggesting that functionally VELCs are closer to a venous phenotype and AELCs are closer to an arterial phenotype^31,32^. Moreover, VELCs showed a slightly higher expression of E-selectin than AELCs, which makes sense taking into account that E-selectin is an adhesion molecule that mediates initial attachment of monocytes^41^. In addition, the response of arterial and venous ECs to vasoactive agents has subtle differences in terms of TEER. For example, thrombin decreases TEER for an extended period in AELCs/HUAECs than in VELCs/HUVECs.

Both AELCs and VELCs show signs of immaturity in response to pro-inflammatory molecules. AELCs or VELCs exposed to TNF-α highly upregulate the expression of ICAM-1 but not the levels of E-selectin or VCAM-1. These results suggest that the iPSC-derived ECs are immature in terms of inflammatory response relatively to somatic ECs. Similar results have been reported previously for ECs derived from hESCs^42^ and from mouse ESCs^43^. In recent studies reporting the derivation of arterial and venous ECs from iPSCs, no characterization was provided about their reactivity to pro-inflammatory molecules^6,13^. However, a previous work in ECs derived from iPSCs in serum-containing medium showed that iPSC-derived ECs were competent as somatic ECs in the response to pro-inflammatory molecules^44^. It is possible that the functional immaturity of our EC subtypes might be connected with the differentiation conditions in the absence of serum and thus additional factors may be needed for the terminal differentiation of the cells. Yet, the inflammatory immaturity of AELCs and VELCs may be an opportunity in the context of cell transplantation. Indeed, the low inflammatory response of AELCs or VELCs may facilitate their integration with the host tissue as observed in previous vascular grafts^45^. In addition, an attenuation of vascular inflammation may decrease the risk of vascular calcification^46^, a risk associated to the vascular grafts^47^; however, this issue deserves further investigation.

Blood contact with polymeric synthetic materials typically leads to pro-coagulant or pro-inflammatory responses and also to ECs activation^48^. Our results show that the PCL and PDMS nanofilms did not induce a pro-inflammatory profile in iPSC-derived ECs and somatic ECs. In addition, we also found that neither PCL nanofilms nor iPSC-derived AELCs (alone or together) induced CD62P (platelet surface P-selectin) translocation to platelets surface in human blood after incubation with those surfaces. P-selectin cross-links platelets and leukocytes and is a major mediator of platelet-leukocyte aggregate formation, thereby upregulating release of proinflammatory cytokines and adhesion to endothelium^40^. These data suggest that both nanofilms and iPS-derived ECs used in this study are hemocompatible.

iPSC-derived ECs adhered to nanofilms, proliferated and formed a monolayer. In terms of cell adhesion, AELCs and VELCs showed lower adhesion in the nanofilms than in TCPS. This was probably due to the lower elastic modulus value of the nanofilms (approximately 1 MPa) in comparison with TCPS (in the order of GPa)^49^. Previous studies suggest that for some cell types cellular adhesion is reduced on softer substrates^50^. In terms of cell viability and proliferation, iPSC-derived ECs showed similar behavior on nanofilms and TCPS. Regarding cell monolayer formation, iPSC-derived ECs were able to organize in a monolayer on top of the nanofilms, which is important for their barrier function. We found that VE-cadherin expression, measured by analysis of fluorescence mean intensity, was higher in ECs cultured in PCL nanofilms. Since VE-cadherin plays a role in the cohesion of intercellular junctions, this suggests that cells cultured on top of the PCL nanofilms have ticker junctions in comparison to the ones formed on PDMS nanofilms.

In conclusion, we have derived arterial- or venous-like ECs in chemically defined conditions by the modulation of VEGF signaling pathway. We further show that these cells share functional properties of somatic ECs such as response to vasoactive agents and do not induce platelet coagulation; however, they have significant differences in their inflammatory profile, either in monoculture or after culture in a scaffold with mechanical properties similar to blood vessels.

## Methods

A Supplementary materials and methods section is provided in Supplementary Information.

### Cell lines and culture conditions

Both iPSCs (K2-iPSC, derived from human cord blood)^46^ and hESCs (clone H9, from WiCell) were cultured in standard conditions (see supplementary information) before differentiation studies. HUVECs (Lonza), HUAECs (Lonza) were used between passage 2 (P2) and passage 4 (P4) HUVECs where acquired from Lonza at P1 and P2, HUAECs at P2. HCAECs (Innoprot) were used at passage P1 and P2.

### Differentiation of hiPSCs into ECs

hiPSCs were incubated with collagenase type IV (1 mg/ml; Gibco) for 45–60 min, cell aggregates were washed in culture medium, mechanically dissociated and plated in fibronectin-coated (Calbiochem, Merck chemicals) petri dishes with a chemically-defined medium (CDM)^23^: Iscove’s Modified Dulbecco’s Medium (IMDM) (Gibco)- Ham’s F12 Nutrient Mixture (F12) (Gibco, [1/1 (v/v)], BSA fraction V (5 mg/ml; Sigma), insulin (7 mg/ml; Sigma), transferrin (15 mg/ml; Sigma) and Pen/Strep (50 U/ml, Lonza). The following supplements were added to CDM according to the scheme in Supplementary Fig. 1: bone morphogenetic protein 4 (BMP-4; 10 or 50 ng/ml; Peprotech), fibroblast growth factor-β (FGF-β; 20 ng/ml; Peprotech), vascular endothelial growth factor 165 (VEGF; 50 ng/ml; Peprotech) and thymosin β4 (Tβ4; 100 ng/ml; Caslo peptide synthesis)^3,20^. After 10 days of culture, cells were harvested with non-enzymatic cell dissociation buffer (Gibco, Life Technologies) and labeled with an antibody against CD31 conjugated with magnetic beads (Miltenyi Biotec). Labeled cells were separated from CD31^−^ populations using a LS column (Miltenyi Biotec), followed by a second purification step using a MS column (Miltenyi Biotec). CD31^+^ enrichment was confirmed by flow cytometry analyses using a different anti-CD31 antibody (WM59; eBiosciences). Isolated cells (passage 0) were initially plated in 24 well plates (30–50,000 cells per well) coated with 0.1% gelatin with endothelial serum free medium (ESFM) (Life Technologies) supplemented with the TGF β-inhibitory molecule SB431542 (10 µM; Tocris) and VEGF (10 or 50 ng/ml) for 4 passages.

Methods for flow cytometry, immunofluorescence, qRT-PCR analyses, endothelial cell function (capillary-like networks on Matrigel and uptake of Dil-Ac-LDL) may be found in detail in Supplementary Materials and Methods section in Supplementary Information.

### Quantification of COUP-TFII positive cells in iPSC-derived ECs

COUP-TFII expression was monitored by immunofluorescence in iPSC-derived ECs, HUVECs (positive control) and HUAECs (negative control). Image acquisition was performed in a High Content Analysis (HCA) microscope (GE Healthcare, Life Sciences) (See Supplementary Methods).

### Monocyte- EC adhesion assay

HUVECs, HUAECs and iPSCs derived-ECs were cultured until confluence. Cells were treated with TNF-α (10 ng/ml, Peprotech) for 6 h, afterwards, cells were co-cultured with CFSE loaded THP1 cells (See Supplementary Methods).

### Expression of ICAM-1, E-Selectin and VCAM-1

HUVECs, HUAECs and iPSCs derived-ECs were treated with TNF-α (10 ng/ml, Peprotech) for 24 h and analyzed by flow cytometry (See Supplementary Methods).

### Intracellular Ca^2+^ variation measurements

HUVECs, HUAECs and iPSC-derived ECs were incubated with acetoxymethyl (AM, 10 μM) derivative FURA-2/AM (1 mM in DMSO, Molecular Probes). Fluorescence measurements in response to thrombin or prostaglandin E2 were done using a microplate fluorescence reader (See Supplementary Methods).

### Trans-endothelial electrical resistance (TEER) measurements

Cells were plated in Matrigel (1:48) coated polycarbonate membrane transwell® inserts (Corning, 3401). The resistance of the monolayer was measured by a Millicell ERS-2 Voltohmmeter (Merck Millipore) after the addition of the stimuli (See Supplementary Methods).

### Nitric oxide (NO) production assay

The ECs were seeded on a 0.1% gelatin-coated 24-well plate (1 × 10^5^ cells/well). For this experiment AELCs and VELCs were maintained in EGM2 (Lonza) medium supplemented with SB431542 (10 µM; Tocris) and VEGF (50 or 10 ng/ml, respectively) until confluence. HUVECs were cultured in EGM-2 medium (Lonza). HCAECs were cultured in endothelial cell growth medium MV-2 (Promocell). Three days later, the media of all four cells was changed to fresh DMEM, low glucose, pyruvate, no glutamine, no phenol red (Gibco®, 11880-036) media containing DAF-FM (1 µM, Life Technologies). Cells were cultured for 30 min and then incubated with fresh media for an additional 15 min to allow complete de-esterification of the intracellular diacetates. Cells were then harvested for flow cytometry analysis. DAF-FM is non-fluorescent until it reacts with NO to form a fluorescent benzotriazole. The flow cytometry analyses were done in a BD Accuri C6 and the data analyses performed by FlowJo_V10 software.

### Cell culture under flow

A suspension of cells (30 µL, 5.5 × 10^5^ cells/channel) was added to each channel of a microfluidic system (µ-Slides, Ibidi) and allowed to flow inside by capillary force. After 4 h, a confluent cell layer was formed, which was then perfused with medium at a flow rate of 5 or 12 dyn/cm^2^, using an Ibidi pump system (perfusion set RED, µSlide VI 0.4). AELCs and VLECs were cultivated in ESFM (Life Technologies) supplemented with SB431542 (10 µM; Tocris) and VEGF (50 or 10 ng/ml, respectively). HUVECs and HUAECs were cultured in EGM-2 (Lonza). Cells were fixed and immunostained for VE-cadherin after 24 h of perfusion. The width and length of the cells were measured by Image J software.

### Preparation of nanofilms based scaffolds

Nanofilms were prepared from poly(caprolactone) (PCL, Mw = 80,000, Sigma), poly(L-lactic acid) (PLLA, Mw = 60,000, Sigma) and poly(dimethylsiloxane) (PDMS, Sylgard® 184 Silicone elastomer kit, Dow Corning). PCL and PLLA were dissolved in chloroform at different concentrations (10, 20 and 50 mg/ml). PDMS was mixed in different proportions with the curing agent [1/10, 1/20 and 1/30 (w/w)]. Nanofilms were prepared in two steps and immobilized in cell crown inserts (Scaffdex) to allow cell culture (see Supplementary Methods).

See Supplementary information for Methods for the characterization of nanofilms.

### Cell counting in nanofilms

4 h after cell plating, cells were stained with Hoechst (0.25 µg/ml in PBS, Molecular Probes for 15 min. Cells were then washed (2x) and maintained in medium. Cells were visualized under a fluorescence microscope and images were acquired using the AxoVision software. Each experimental condition was analyzed in triplicate and a minimum of 6 fields were acquired. Cell nuclei were counted using the ImageJ.

### Presto Blue cell viability assay

Cells cultured for 4 or 72 h were incubated with Presto Blue cell viability reagent (Molecular Probes) for 2 h at 37 °C. Fluorescence (excitation: 560 nm; emission: 590 nm) was measured by a microplate reader (SYNERGY H1, BIOTEK).

### Effect of nanofilms and iPSC-derived ECs on platelet activation

Human peripheral blood was collected (with donors informed consent) in citrate tubes and, in less than 1 h, diluted with Tyrode’s buffer (TB) (10 mM HEPES, 137 mM NaCl, 2.8 mM KCl, 1 mM MgCl_2_, 12 mM NaHCO_3_, 0.4 mM Na_2_HPO_4_, 0.35% (w/v) BSA and 5.5 mM glucose) in the ratio of 1:10, at room temperature. The diluted blood (500 μL) was added to the cells and/or surfaces and incubated for 90 min at 37 °C. After incubation, samples (25 μL) were transferred to cytometer tubes containing TB (50 μL), CD61-PerCP (BD Biosciences) (20 μL, for platelet discrimination) and CD62P-PE (BD Biosciences) (20 μL, for platelet activation). Thrombin (10 UI/ml, Sigma–Aldrich) was used as a positive control. All samples were incubated for 30 min at RT, in the dark. After incubation, TB (500 μL) was added to each sample. Afterwards, samples were fixed with 2% paraformaldehyde/PBS and kept at 4 °C until they were analyzed with the flow cytometer.

### Statistical analyses

Results were analyzed by GraphPad Prism 6 (version 6.01) (GraphPad Software, La Jolla USA) software. We performed D’Agostino Pearson omnibus normality test and Shapiro-Wilk normality test in GraphPad. The results that did not pass normality test and/or with low sampling were analyzed using a non-parametric test. Sample pairs were analyzed with the Mann-Whitney’s test. Multiple samples were evaluated by Kruskal-Wallis test followed by a Dunn’s multiple comparison’s test (unless otherwise stated) to evaluate the statistical differences (P ≤ 0.05) among all samples or between samples and controls, respectively. All error bars are standard error of the mean (SEM).

## Supplementary information

Supplementary information  online

## Data Availability

The datasets generated during and/or analyzed during the current study are available from the corresponding author on reasonable request.
