# Supplementary material for: Functional characterization of iPSC-derived arterial- and venous-like endothelial cells
Source: Sci Rep. 2019 Mar 7;9:3826. doi: 10.1038/s41598-019-40417-9 (PMC6405900; doi:10.1038/s41598-019-40417-9)
Supplement: Supplementary file 1 — Supplementary information online [file 41598_2019_40417_MOESM1_ESM.pdf]

Title Page

Title: Functional characterization of iPSC-derived arterial- and venous-like endothelial cells

S. Rosa [1], C. Praça[1,4], P. R. Pitrez[1,4], P. José Gouveia [1,2], X. L. Aranguren[5], L. Ricotti [3], L. Silva Ferreira [1,4,]*

[1] CNC-Center of Neurosciences and Cell Biology, University of Coimbra; 3004-517 Coimbra, Portugal

[2] Instituto de Investigação Interdisciplinar, University of Coimbra, Casa Costa Alemão - Pólo II, Rua Dom Francisco de Lemos, 3030-789, Coimbra, Portugal

[3] The BioRobotics Institute, Scuola Superiore Sant' Anna, Viale Rinaldo Piaggio 34, 56025 Pontedera (PI), Italy

[4] Faculdade de Medicina da Universidade de Coimbra, 3000-354 Coimbra, Portugal

[5] Hematology and Cell Therapy Area, Clinica Universidad de Navarra, and Division of Oncology, Center for Applied Medical Research, University of Navarra, Pamplona, Spain

* Corresponding author (Email: lino@uc-biotech.pt)

Running Title: **Function of iPSC-derived endothelial cell subtypes**

Supplementary Information:

1. Supplementary Materials and Methods Section
2. Supplementary Data References
3. Supplementary table S1- List of antibodies used for immunofluorescence (ICC) and flow cytometry (FC) analyses
4. Supplementary table S2- Primer sequences for qPCR analyses.
5. Supplementary Figures (1 to 10) and respective Figure Legends

Supplementary Materials and Methods Section:

Cell lines and culture conditions. Both iPSCs (K2-iPSC, derived from human cord blood)[1] and hESCs (clone H9, from WiCell) were cultured in standard conditions. Pluripotent stem cells were co-cultured with mytomicin-inactivated mouse embryonic fibroblasts (MEFs) (Global Stem) in hPSCs medium: 80% KO-DMEM (Gibco), 20% KO-serum (Gibco), 1 mM glutamine (Life Technologies), 4 ng/ml β-FGF (Peprotech), 0.5 mM β-mercaptoethanol (Sigma), 1x MEM non-essential aminoacids (MEM NEAA; Gibco) and 50 U/ml Pen/Strep (Lonza). HUVECs (Lonza), HUAECs (Lonza) were used between passage 2 (P2) and passage 4 (P4). HCAECs (Innoprot) were used at passage P1 and P2.

Flow cytometry analyses. hiPSCs-derived ECs, HUVECs and HUAECs were dissociated with non-enzymatic cell dissociation buffer (Gibco) for 10 min, followed by gentle pipetting and washes in PBS with 5% FBS. Single cells were aliquoted in PBS with 5% FBS (between 100,000 and 125,000 cells were used per condition) and stained with either isotype controls or antigen-specific fluorescent-conjugated antibodies for 30 min at 4ºC. For VE-Cadherin and vWF analysis cells were fixed with 1% paraformaldehyde (PFA) for 10 min, permeabilized with Triton 0.1% for 10 min, and were incubated with primary antibody for 1 h at room temperature followed by the secondary antibody for 30 min at room temperature. For ICAM-1 analysis cells were incubated with primary antibody for 1 hour at 4ºC, followed by the secondary antibody for 30 min at room temperature. The antibodies utilized and respective dilutions can be found in **Supplementary Table S1**: *List of antibodies used for immunofluorescence (ICC) and flow cytometry (FC) analyses* . The flow cytometry analyses were performed in BD FACSCalibur and BD Accuri C6, data analysis was performed with FlowJo_V10. Ten thousand events were collected in each run. The percentages showed in the dotplots were calculated based on the isotype controls represented by light blue. Isotype controls had 1% overlap with the protein of interest.

Immunofluorescence analyses. Cells were fixed with 4% (v/v) paraformaldehyde (PFA) for 10 min. When necessary, cells were permeabilized with 0.1% (v/v) Triton in PBS (10 min) and blocked with 1% (w/v) BSA solution for 30 min at room temperature. Antibodies were diluted with antibody diluent background reducing solution (DAKO). Cells were incubated for 1 h at room temperature with the primary antibodies, washed with PBS (3x5 min washes), incubated with a secondary antibody for 30 min at room temperature and thereafter counterstained with DAPI. The antibodies used and respective dilutions are found in **Supplementary Table S1**. Immunofluorescence analyses were performed on a confocal LSM 710 microscope (Zeiss).

Quantification of COUP-TFII positive cells in iPSC-derived ECs- COUP-TFII expression of protein was monitored by immunofluorescence in iPSC-derived ECs, HUVECs (positive control) and HUAECs (negative control). Image acquisition was performed in a In Cell Analyser High Content Analysis (HCA) microscope (GE Healthcare, Life Sciences). Image analyses were performed with the In Cell Investigator software (Image analysis software for IN Cell analyser systems). The nuclei presenting an intensity value equal or higher than the intensity value of HUAEC nuclei (289.6±105.9 arbitrary units) plus twice the standard deviation value (500 arbitrary units) were considered positive. For each well, images were acquired in 6 different fields. Each field contained 300 cells in average.

Quantification of Ephrin B2 positive iPSCs-derived ECs. Expression of Ephrin B2 protein was monitored by immunofluorescence in iPSCs-derived ECs, HUAECs (positive control) and HUVECs (negative control). Image acquisition was performed with a Confocal LSM 710 microscope (Zeiss). Image analyses were performed with Image J software. The cells presenting an intensity value equal or higher than twice the average intensity value of Ephrin B2 in HUVECs (61.3 arbitrary units) were considered positive. For each cell type approximately 100 cells were counted.

Total RNA extraction and quantitative real-time polymerase chain reaction (qRT-PCR) analyses. Total RNA was extracted with Trizol reagent (Invitrogen) and immediately stored at -80ºC. RNA was quantified in a NanoDrop ND-1000 spectrophotometer (NanoDrop Technologies,

Inc., USA) at 260 nm. The cDNA was reverse transcribed from 1 µg of total RNA using TaqMan reverse transcription reagents kit (Invitrogen) according to the manufacturer instructions. The cDNA obtained was stored at -20ºC until further analysis by real-time PCR was performed. When only a small number of cells was available for RNA extraction, Ambion Cells to Ct Kit (Life Technologies) or RNEASY micro kit (QUIAGEN) was used accordingly to the manufacturer instructions. Real-time PCR analyses were performed by using the fluorescent dye SYBR green (Applied Biosystems) and the 7500 Fast Real Time PCR System (Applied Biosystems). Specific sets of primers were designed by SIGMA. Sequences of the primers are in **Supplementary Table S2**.

Capillary-like networks formation on Matrigel. A 96 Multi well plate was coated with Matrigel (BD) (50 µL/well – Matrigel was thawed at 4ºC and both tips and plates were kept cold during the procedure) and placed at 37 ºC for 30 min. Cells were seeded on top of the polymerized Matrigel at a density of 4-10x10^3 cells/well in 100 µL of EGM-2 (Lonza) medium. After 6 h, bright field images were acquired in InCell Analyser HCA System, and capillary-like networks analyzed using ibidi ACAS image analysis.

Dil-Ac-LDL Uptake. Dil-Ac-LDL (Harbor Bio-Products) was added to the cell culture medium (20µg/ml) and cells maintained at 37ºC for 4h. In the last 10 min of incubation, Hoechst dye (0.25µg/ml) was added to stain cel nuclei. Cells were then washed and maintained with medium. Images were acquired in the InCell Analyser HCA System and analyzed by the corresponding software.

Monocyte- EC adhesion assay. HUVECs, HUAECs and iPSCs derived-ECs were cultured in 24-well plates until confluence. The cells were then treated with TNF-α (10 ng/ml, Peprotech) a pro-inflammatory cytokine, for 6 h. In some wells, cells were counted after trypsinization to obtain the average number of cells per well. After 6 h, cells were washed 3 times in culture medium and co-cultured with monocytes at the ratio of 1:2 for 30 min. The human monocyte cell line THP1 was used. These cells were previously loaded with the fluorescent dye CFSE (Molecular Probes) according to manufacturer instructions. Briefly, THP1 (1×10^6 cells/ml) were incubated with CFSE (5 µM) for 15 min at 37ºC and washed three times in culture medium to remove the unbound dye. After 30 min, cells were washed 3 times with PBS to remove unbound THP1 cells, nuclei were stained with Hoechst (Molecular Probes), and the images were acquired in the InCell Analyser HCA System (GE Healthcare) and analyzed by the corresponding software. For non-treated and TNF-α-treated cells the percentage of THP1 cells was calculated relative to the total number of nuclei counted for each field. Each condition was performed in triplicate and a minimum of 12 fields were acquired per each well; approximately 300 cells were counted per each field.

Intracellular Ca2+ variation measurements. HUVECs, HUAECs and iPSC-derived ECs were incubated with a membrane permeable acetoxymethyl (AM, 10 µM) derivative FURA-2/AM (1 mM in DMSO, Molecular Probes) and 0.06% (w/v) Pluronic F-127 (Sigma) diluted in endothelial serum free medium (without antibiotics) (Life TechnologiesA volume of 50 µl/well was used and cells were placed in the incubator for 1 h. The medium was then replaced by the respective basal medium and cells were further incubated for 30 min. Cells were washed twice with 100 µL sodium salt solution (140 mM NaCl, 5 mM KCl, 1 mM CaCl2, 1 mM MgCl2, 10 mM Glucose, 10 mM HEPES-Na+ pH 7.4). The buffer was then replaced again (100 µl/well) immediately prior to incubating or not with test compounds. Cells were incubated with thrombin (5 U/ml, Sigma, T7009) or prostaglandin E2 (PGE2, 200 ng/ml, Cayman Chemical, 14010). Fluorescence was measured at emission 510 nm using two alternating excitation wavelengths (340 nm and 380 nm) using the microplate fluorescence reader (Spectramax Gemini EM, Molecular Devices). Each well was read every 6 s.

Trans-endothelial electrical resistance (TEER) measurements. Cells were plated in polycarbonate membrane transwell® inserts, pore size 0.4 µm for 12 well culture plates (Corning, 3401). Culture inserts were coated previously to the seeding of the cells with Matrigel (BD, 354234) diluted with DMEM (1:48) for 1 h. Cells were seeded at a density of 0.8 $\times10^5$ by insert and were left

to stabilize for 3 days, after which thrombin (5 U/ml, Sigma, T7009) or PGE2 (200 ng/ml, Cayman, 14010) were added. The resistance of the monolayer was measured by a Millicell ERS-2 Voltohmmeter (Merck Millipore) after the addition of the stimuli. An empty filter was used to determine the background resistance. Three separate filters were used for each condition (type of cell), and the mean resistance was calculated after background subtraction. TEER measurements were performed again 48 h after the end of the experiment to verify the maintenance/or stability of the measured resistance values for each monolayer evaluated.

Preparation of nanofilms based scaffolds. Nanofilms were prepared from poly(caprolactone) (PCL, Mw=80,000, Sigma, 440744-0250), poly(L-lactic acid) (PLLA, Mw=60,000, Sigma, 81273) and poly(dimethylsiloxane) (PDMS, Sylgard® 184 Silicone elastomer kit, Dow Corning, DLE-1673921). PCL and PLLA were dissolved in chloroform at different concentrations (10, 20 and 50 mg/ml). PDMS was mixed in different proportions with the curing agent [1/10, 1/20 and 1/30 (w/w)]. Nanofilms were prepared in two steps. Initially, a sacrificial layer of poly(vinyl alcohol) (1% (w/v), average Mw = 25,000, 88% hydrolysed, approximately 2 ml per sample) was deposited by spin coating on a silicon wafer (400 μm thick, 2 cm × 2.5 cm; Primewafers) at 4,000 rpm for 20 sec. Then, a nanofilm was obtained by spinning the polymer solution using the same spinning parameters. Dissolution of the sacrificial layer and consequent release of the insoluble freestanding nanofilm was achieved by immersing the polymer-coated silicon wafer in water. The freestanding nanofilms were then immobilized in cell crown inserts (Scaffdex, Z681881) to keep them firmly fixed during cell culture. Nanofilms were sterilized by incubation with a solution of Pen/Strep (500 U/ml, Lonza, LONZ17-602E) containing ciprofloxacin (100 μg/ml, Sigma, 17850) and amphotericin B (6.25 g/L, Sigma, A9528) in PBS for approximately 16 h. After extensive (5 ×) washes in PBS, nanofilms were coated with 0.1% (w/v) gelatin (porcine origin, Sigma, G1890).

Characterization of nanofilms: mechanical tests and atomic force microscopy (AFM) analyses. The mechanical properties of the nanofilms was evaluated by measuring their strain in response to an applied unidirectional stress. An Instron 4464 mechanical testing system equipped with a ±10 N load cell was used. Traction tests were performed on ten samples for each group. The nanofilms were detached from the substrate, by immersing them in water, then gently fished up and allocated between two aluminium clamps. All specimens were pulled at a constant speed of 5 mm/min until sample failure was reached. Data were recorded at a frequency of 100 Hz. The stress was calculated as the ratio between the load and the cross-section area of a tensile specimen, while the strain was calculated as the ratio between its extension and its initial length. The Young's modulus for each tested sample was extracted from its stress/strain curve, according to a standard procedure[2]. Imaging was performed with a Veeco Innova scanning probe microscope (Veeco Instruments Inc., Santa Barbara, CA) in dry state, operating in tapping mode, with oxide-sharpened silicon probes (RTESPA-CP, Veeco Instruments Inc.) at a resonant frequency of ~300 kHz. Each sample was gently scratched at its surface and then scanned across the scratch edge, over a 50 μm × 50 μm area, recording 128 × 128 values per each image. The resulting scan data were elaborated using the Gwyddion SPM analysis tool (http://gwyddion.net). They were levelled with the facet level tool to remove sample tilt, then the film thickness was evaluated as the difference between the average heights of a region of interest (ROI) selected on the nanofilm surface and the average height of the ROI on the silicon wafer. The analysis tool also quantified the average surface roughness for each image. For thickness and roughness measurements, three different images were acquired for each sample and three independent samples were analyzed for each sample type.

Expression of ICAM-1, E-Selectin and VCAM-1 by flow cytometry. HUVECs, HUAECs and iPSCs derived-ECs were cultured in 24-well plates until confluence. The cells were then treated with TNF-α (10 ng/ml, Peprotech, 300 01A) for 24 h and finally harvested using a Tryple Select

solution (Gibco,12604-021). Approximately 100,000 to 125,000 cells were stained with anti-human E-selectin (CD62E)-PE conjugated or anti-human VCAM-1 FITC-conjugated, for 30 min at 4ºC. For ICAM-1 (1:50; Santa Cruz) cells were incubated for 1 h at 4ºC, followed by incubation with the secondary antibody for 30 min. The antibodies used as well as the dilutions may be found in Supplementary Table I- List of antibodies used for immunofluorescence (ICC) and flow cytometry (FC) analyses. The flow cytometry analyses were done by a BD FACSCalibur or a BD Accuri C6 while data analysis was performed with FlowJo_V10.

Supplementary References:

1 Haase, A. *et al.* Generation of Induced Pluripotent Stem Cells from Human Cord Blood. *Cell Stem Cell* **5**, 434-441 (2009).

2 Callister. *Materials Science and Engineering: An Introduction* 6th edn, pp 113–52 (New York: Wiley, 2003).

Supplementary table S1- List of antibodies used for immunofluorescence (ICC) and flow cytometry (FC) analyses

Antibody	Application	Dilution	Amount (µg)	Reactivity	Host	Vendor	Catalog No.
IgG1K – FITC IC	FC	1:20 100µl	0.5µg	Human	Mouse	e-Biosciences	11-4714-81 clone:P3.6.2.8.1
CD31-FITC	FC	1:20 100µl	0.5µg	Human	Mouse	e-Biosciences	11-0319-42 clone: WM-59
IgG1-PE IC	FC	10µl 100µl	-	Human	Mouse	R&D	IC002P clone:11711
KDR-PE	FC	10µl 100µl	-	Human	Mouse	R&D	FAB357P clone: 89106
VE-Cadherin	FC/ICC	1:50 100µl	0.4µg	Human	Mouse	Santa Cruz	sc-9989 clone: F8
EphB2-PE	FC	10µl 100µl	-	Human and others	Rat	R&D	FAB467P clone: 512012
VCAM1-FITC	FC	1:10 100µl	5ug	Human	Mouse	BD Biosciences	555645 clone: 51-10C9
IgG2Ak IC-PE	FC	5µl 100µl	0.5µg	Human	Mouse	BioLegend	400213 Clone:MOPC-173
E-selectin-PE	FC	5µl 100µl	0.5µg	Human	Mouse	BioLegend	322605 clone: HCD62E
ICAM-1	FC	1:50 100µl	0.4µg	Human and others	Mouse	Santa Cruz	sc-107 clone: 15.2
CD31	ICC	1:50 100ul	0.4µg	Human and others	Mouse	DAKO	M0823 clone: JC70A
vWF	FC/ICC	1:200 100µl	1.6µg	Human and others	Rabbit	DAKO	A0082
KDR	ICC	1:100 100µl	0.5µg	Human and others	Rabbit	Abcam	ab234110
COUP-TFII	ICC	1:200 100µl	0.5µg	Human	Mouse	R&D	PP-H7147-00 Clone H7147
Ephrin B2	ICC	1:50 100µl	0.4µg	Human and others	Mouse	Santa Cruz	SC-15397 Clone: H-83
Ephrin B2	ICC	1:50 100µl	0.4µg	Human and others	Mouse	Santa Cruz	SC-398735 Clone: F-2
ZO-1	ICC	1:200 100µl	0.125µg	Human and others	Rabbit	Life Technologies	617300
Alexa Fluor 488	ICC/FC	1:200/1:500 100µl	1µg/0.4µg	Mouse	Goat	Life Technologies	A11001
Alexa Fluor 488	ICC/FC	1:200/1:500 100µl	1µg/0.4µg	Rabbit	Goat	Life Technologies	A11034
Alexa Fluor 555	ICC/FC	1:200/1:500 100µl	1µg/0.4µg	Mouse	Goat	Life Technologies	A21422
Anti-rabbit-Cy3	ICC	1:100 100µl	1.5µg	Rabbit	Goat	Jackson Imunoresearch	111-165-144
Anti-	ICC	1:50	2µg	Mouse	Sheep	Sigma	C2181

mouse-Cy3	100μl

Supplementary table S2- Primer sequences for qPCR analyses.

**Gene name**	**Sense (5´-3´)**	**Antisense (5´-3´)**
Brachyury	TGCTTATTTCCGTCCATTTCC	TTACGCACACCCAGGATC
CD31	AGATACTCTAGAACGGAAGG	CAGAGGTCTTGAAATACAGG
KDR	GTACATAGTTGTCGTTGTAGG	TCAATCCCCACATTTAGTTC
VECADH	CGCAATAGACAAGGACATAAC	TATCGTGATTATCCGTGAGG
EFNB1	CAACACTGTCAAGATGGC	CTCTTCTCTTCCTGGTTCA
EFNB2	CCACAGATAGGAGACAAATTG	AGTTGAGGAGAGGGGTAT
JAG1	GTCTCAAAGAAGCGATCAG	ATATACTCCGCCGATTGG
HEY1	CCGGATCAATAACAGTTTGTC	CTTTTTCTAGCTTAGCAGATCC
NOTCH4	ATTGACACCCAGCTTCTTG	GAGGACAAGGGTCTTCAA
EPHB4	CTCAGTTCGGATCCTACC	AATGTCACCCAGTTCAGAT
NR2F2	CAGAACAACTTGCTACTTATCA	GGTACATAGACACAGGACAA
e-SELECTIN	AGCTTCCCATGGAACACAAC	CTGGGCTCCCATTAGTTCAA
ICAM1	CAAGGCCTCAGTCAGTGTGA	CCTCTGGCTTCGTCAGAATC
VCAM1	ACTTGATGTTCAAGGAAGAG	TCCAGTTGAACATATCAAGC
CXCR4	AACTTCAGTTTGTTGGCTG	GTGTATATACTGATCCCCTCC
DLL4	AGGTGTGGAAGGGTATTG	GGAGGTATAAGGCAGGAG
GAPDH	AGCCACATCGCTCAGACACC	GTACTCAGCGCCAGCATCG

Supplementary Figures (1 to 10) and respective Figure Legends

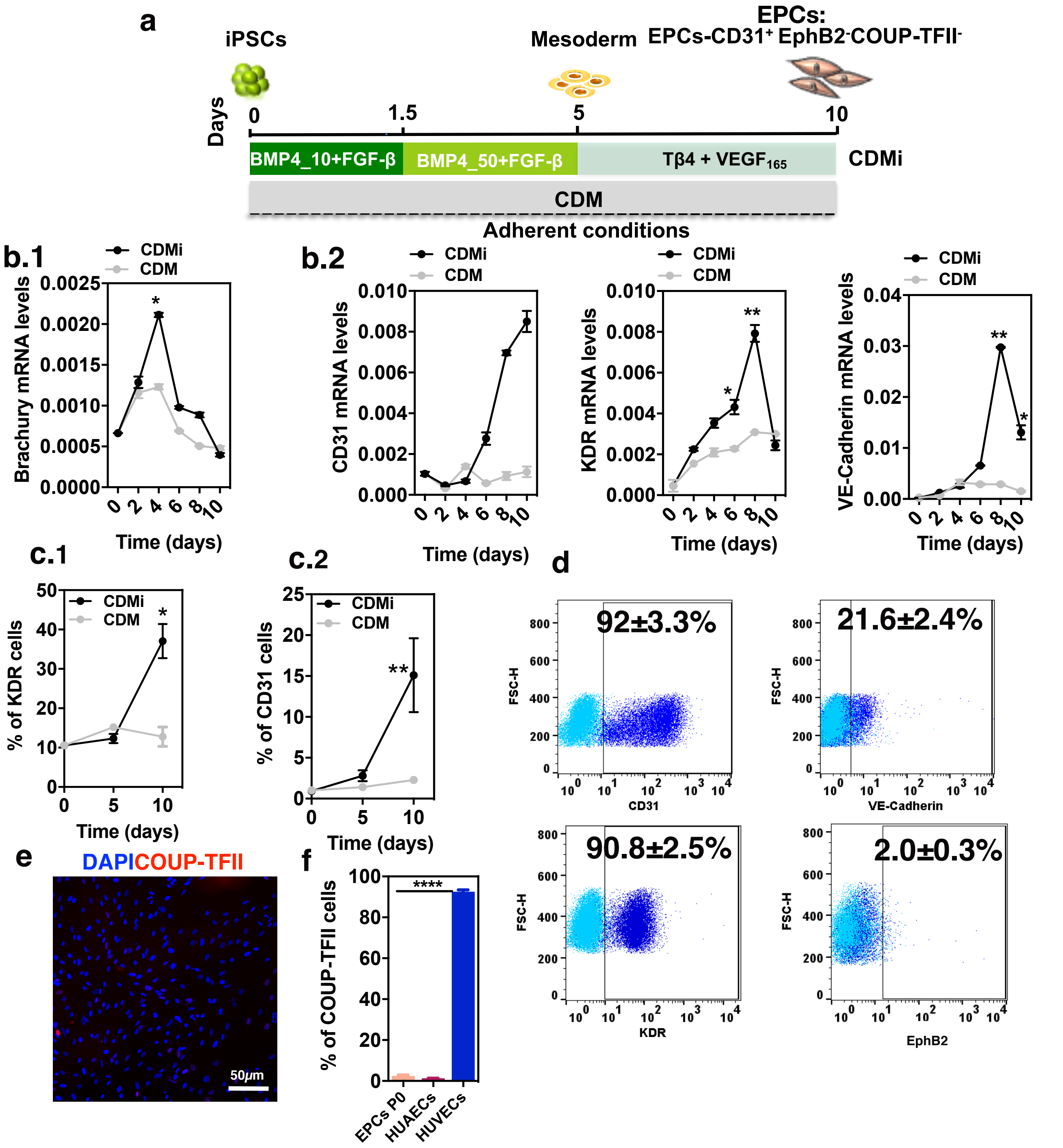

Supplementary Figure S1- Differentiation of hiPSCs into EPCs. (a) Schematic representation of the protocol. hiPSCs colonies were plated in fibronectin-coated plates in CDM supplemented with different inductive agents (CDMi) for 10 days. EPCs were isolated by magnetic labeling (P0) and characterized. **(b)** qRT-PCR of a mesoderm (*Brachyury*, **b.1**) and endothelial genes (*CD31*, *KDR* and *VE-Cadherin*, **b.2**). Data were normalized by the reference gene *GAPDH* and relative to undifferentiated hiPSCs. **(c)** Analyses of CD31 and KDR by flow cytometry. **(d)** Expression of EC markers CD31, VE-Cadherin, KDR and arterial marker EphB2 by flow cytometry in EPCs (P0). **(e)** Expression of venous marker COUP-TFII in EPCs P0 by immunocytochemistry. **(f)** Quantification of COUP-TFII positive cells. Images were acquired in 6 different fields that contained an average of 300 cells. In **b** and **c** statistical analysis were made at each time point between CDM and CDMi and were performed by a Kruskal-Wallis test followed by a Dunn´s post-test. In **f** statistical analyses were performed by ANOVA followed by a Dunnet´s post-test. *p<0.5, **p<0.1, ***p<0.001, ****p<0.0001. In **b**, **c**, **d** and **f** results are mean ± SEM (n=3).

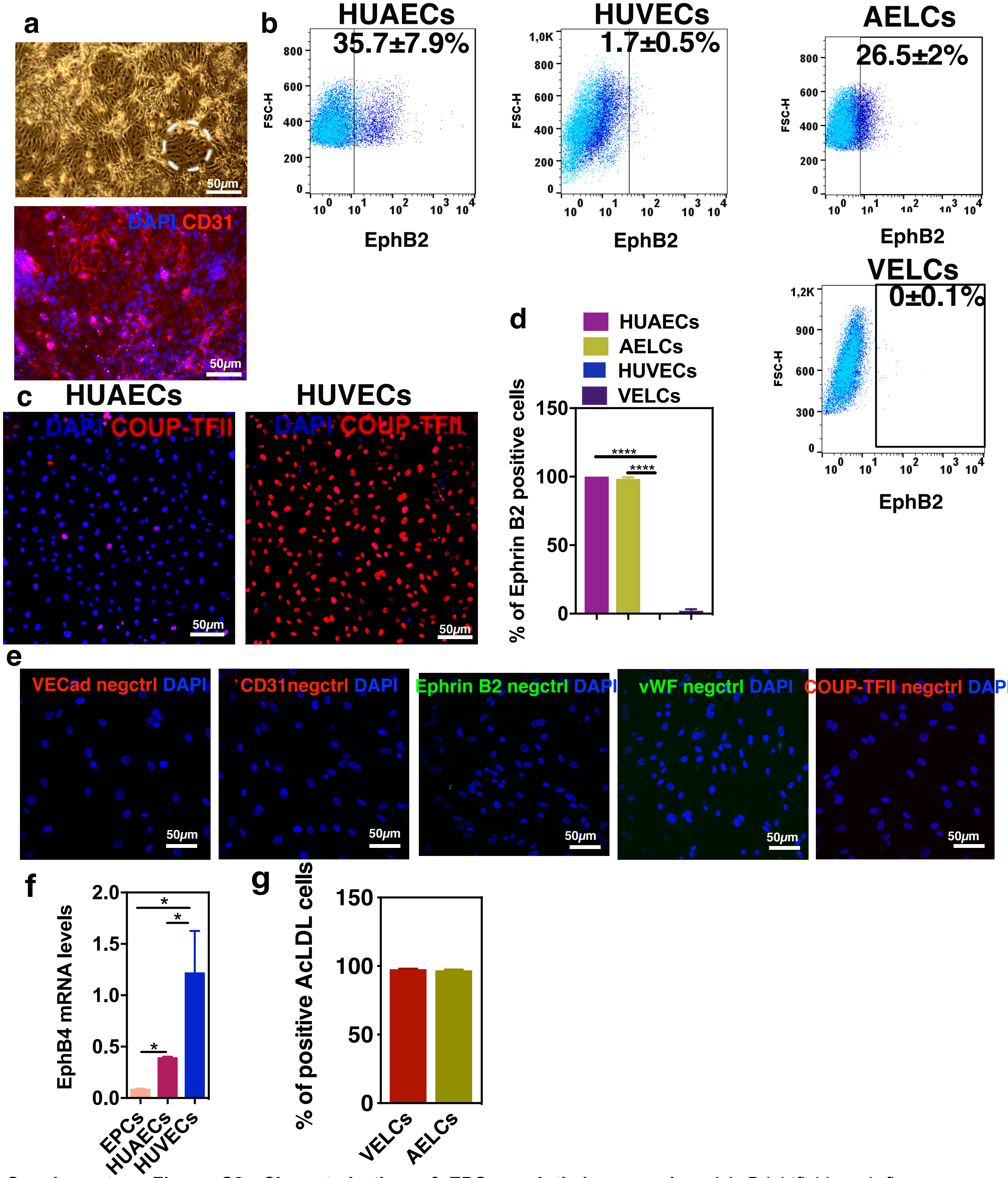

Supplementary Figure S2- Characterization of EPCs and their progenies. **(a)** Brightfield and fluorescence microscope images of endothelial cell islands at day 10 of differentiation. EPCs were labeled for CD31 marker. Bar indicates 50 µm. **(b)** Expression of arterial marker EphB2 in HUAECs, HUVECs, AELCs and VELCs, as assessed by flow cytometry. Results are mean ± SEM (n=3). **(c)** Expression of venous marker COUP-TFII in HUAECs and HUVECs as evaluated by immunocytochemistry. **(d)** Percentage of Ephrin B2 positive cells on AELCs, VELCs, HUAECs and HUVECs. In average approximately 100 cells were counted for each cell type (n=3). **(e)** Isotope controls for VECad, CD31, EphrB2, vWF and COUP-TFII. Bar indicates 50 µm. **(f)** Expression of venous (EphB4) mRNA transcript in HUVECs, HUAECs and EPCs, as evaluated by qRT-PCR analyses. Results are mean±SEM (n=3-6). **(g)** Quantification of AcLDL uptake by AELCs and VELCs. Statistical significance was assessed using Mann Whitney test. *p<0.5, **p<0.1, ***p<0.001, p****<0.0001.

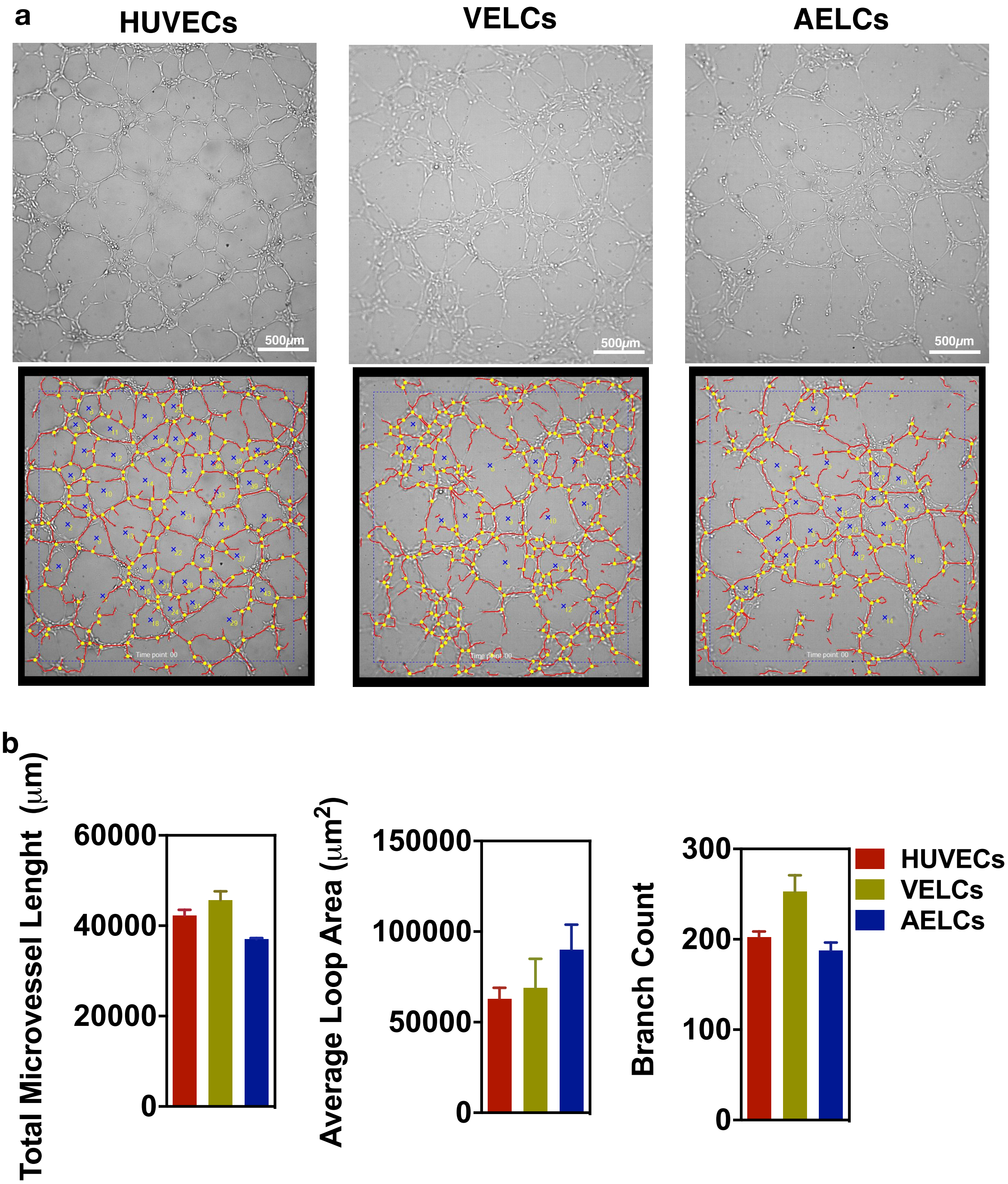

Supplementary Figure S3- Microvascular network from HUVECs, AELCs or VELCs plated on top of Matrigel. (a) Representative brightfield microscope images (4x magnification) of microvascular networks in Matrigel after 6 h and the corresponding structure analyses (tubes-red; branch points-yellow; loops-blue) by the Ibidi ACAS image analysis program. **(b)** Quantification of microvascular network parameters. Results are average ± SEM (n=3). Statistical significance was assessed by Kruskall Wallis, followed by a Dunn´s multicomparison test.

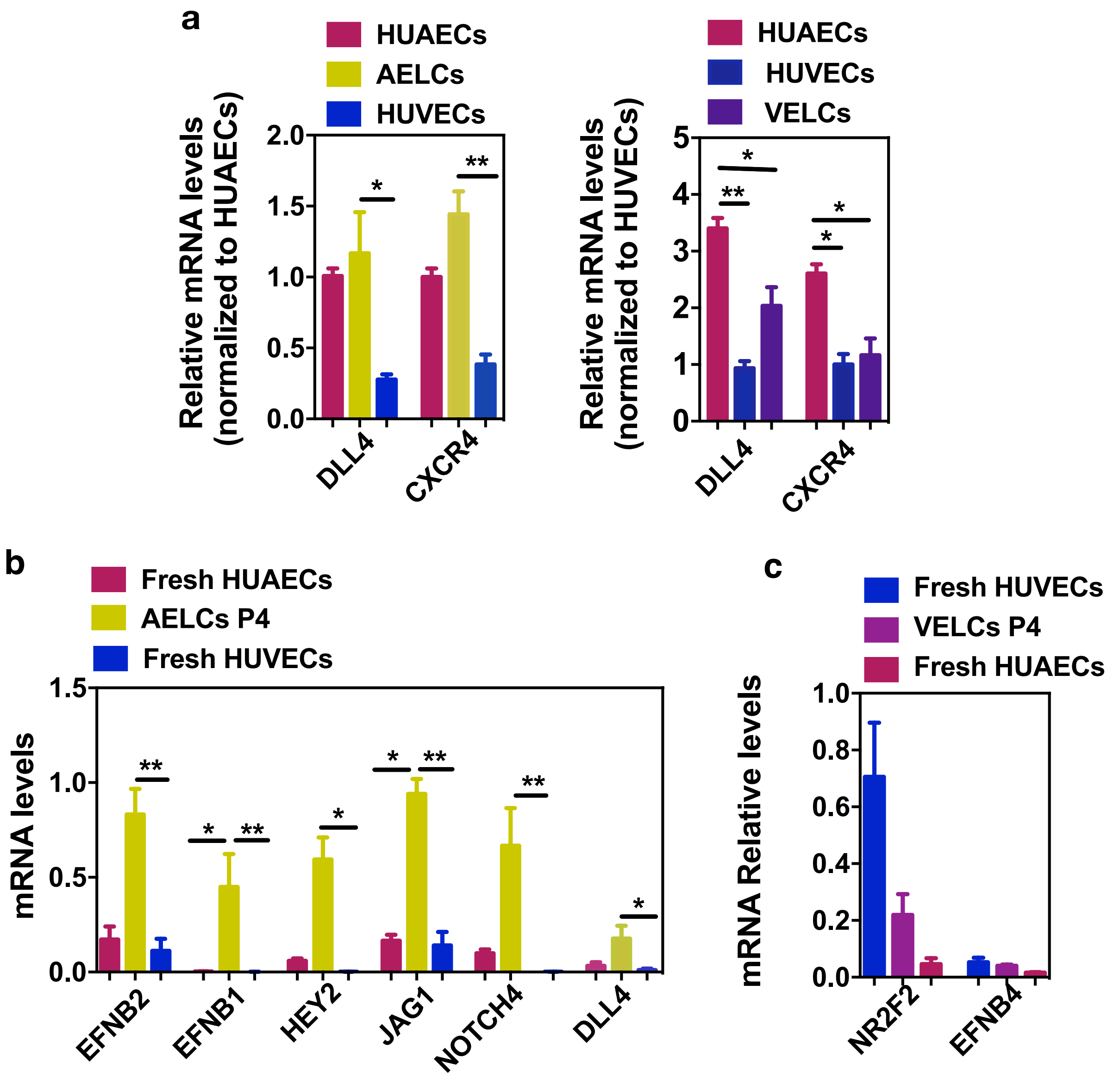

Supplementary Figure S4- Characterization of AELCs and VELCs. **(a)** qRT-PCR of arterial markers DLL4 and CXCR4 on P4 AELCs and VELCs. Results were normalized by GAPDH and expressed relatively to HUAECs and HUVECs between passages P2 to P4 (n=4). **(b)** qRT-PCR of arterial markers on P4 AELCs, fresh HUAECs (P0) and HUVECs (P0). Data was normalized by *GAPDH*. Results are mean ± SEM (n=4). **(c)** qRT-PCR of venous markers on P4 VELCs, fresh HUAECs (P0) and fresh HUVECs (P0). Data was normalized by *GAPDH.* Results are mean ± SEM (n=4). In a-c stastistical analyses were performed using a Kruskal-Wallis test followed by a Dunn's multiple comparisons test. *p<0.5, **p<0.1, ***p<0.001.

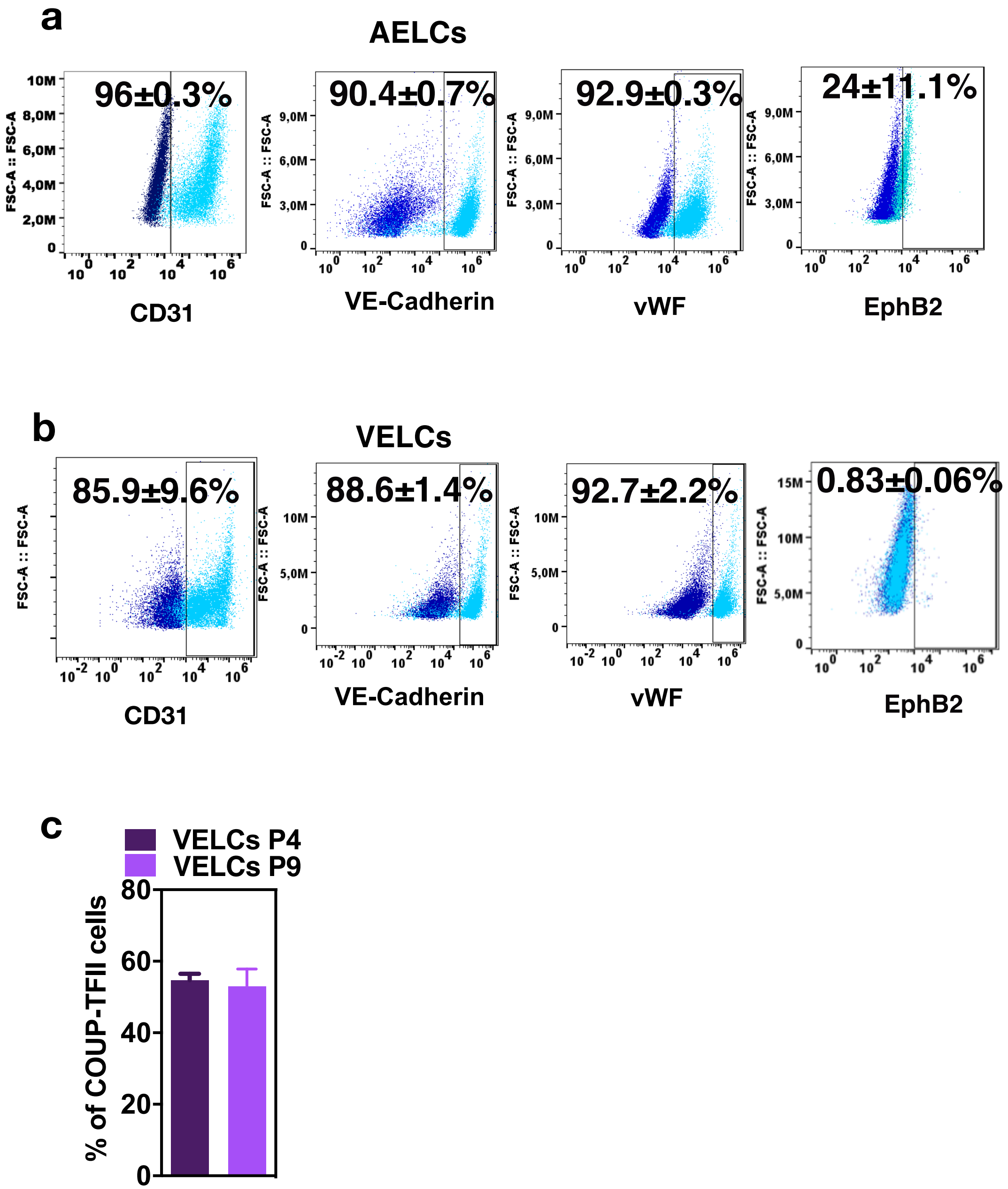

Supplementary Figure S5- Characterization of AELCs and VELCs at passage 9. (a) Flow cytometry analyses of general EC (CD31, VE-cadherin, vWF) and arterial (EphB2) markers on AELCs-P9. **(b,c)** Flow cytometry **(b)** and immunostaining **(c)** analyses of general EC (CD31, VE-cadherin, vWF) and arterial (EphB2) and venous markers (COUP-TFII) on VELCs P9. Results are mean ± SEM (n=3). Statistical analysis was done using a Mann-Whitney test.

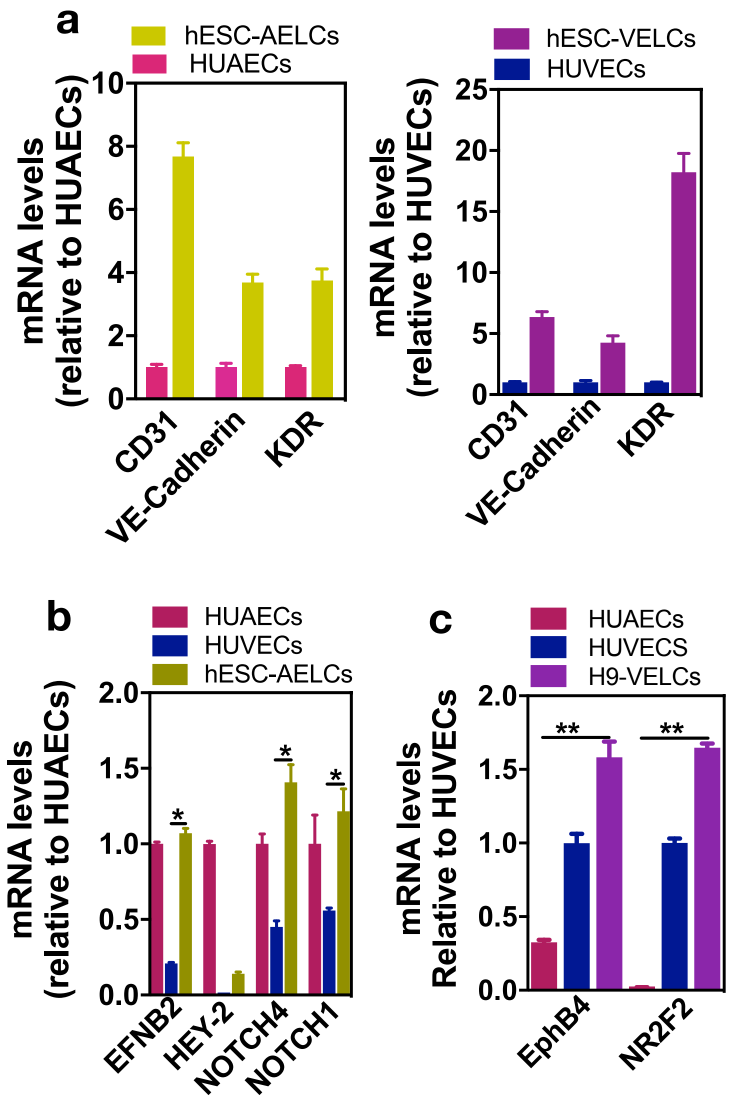

Supplementary Figure S6- Characterization of AELCs and VELCs derived from hESCs. (a) Expression of general EC markers on hESC-AELCs, hESC-VELCs, HUVECs and HUAECs by qRT-PCR analyses. Results are mean ± SEM (n=3). **(b)** Expression of arterial markers on hESC-AELCs, HUAECs and HUVECs. Results are mean ± SEM (n=3). Expression of arterial markers on hESC-AELCs was compared to HUVECs and HUAECs. **(c)** Expression of venous markers on hESC-VELCs, HUVECs and HUAECs by qRT-PCR analyses. Results are mean ± SEM (n=3). Expression of venous markers on VELCs was compared to HUAECs and HUVECs. in **b** and **c**, statistical analyses were performed by a Kruskal-Wallis test followed by a Dunn´s post-test. *p<0.5, **p<0.1.

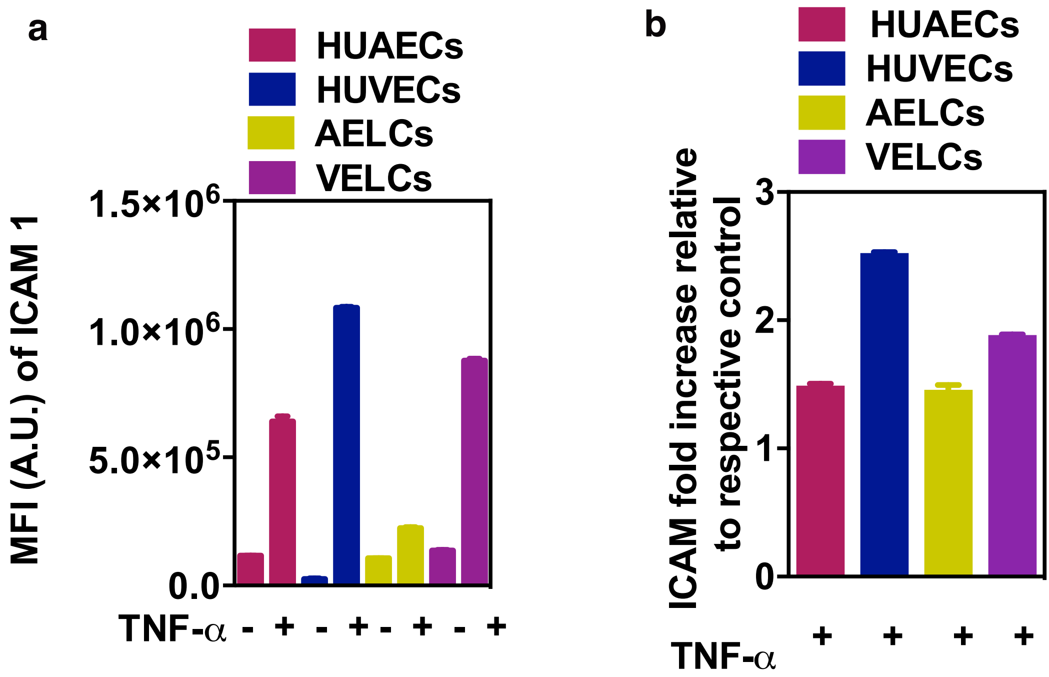

Supplementary Figure S7- Functional characterization of AELCs and VELCs. **(a)** Mean fluorescence intensity of ICAM1 marker, as evaluated by flow cytometry. Cells were exposed to TNFα (10 ng/ml) during 24 h. Results are average ± SEM (n=3). **(b)** ICAM1 mRNA fold increase in HUVECs, HUAECs, AELCs and VELCs after exposure to TNFα (10 ng/ml) during 6 h. In each sample, ICAM1 expression was initially normalised by GAPDH expression followed by the normalisation with ICAM1 expression in cells not exposed to TNFα. Results are average ± SEM (n=3).

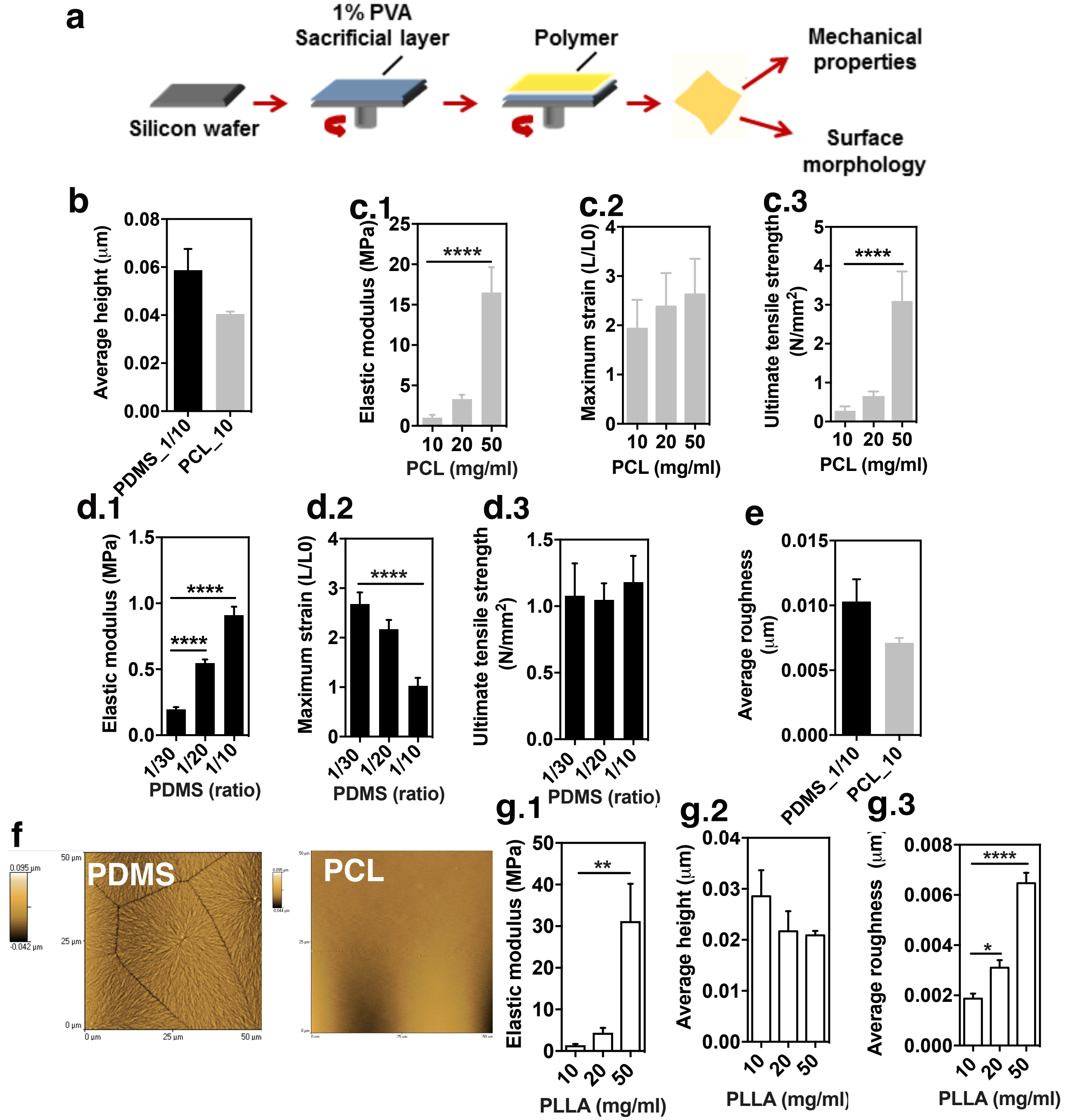

Supplementary Figure S8- Characterization of nanofilms. (a) Schematic representation of the protocol for the preparation of PCL and PDMS nanofilms. **(b)** Average height of PCL (10 mg/mL) and PDMS (1/10) nanofilms as evaluated by AFM analyses. Results are mean ± SEM (n=9). **(c and d)** Mechanical properties of PCL **(c)** and PDMS **(d)** nanofilms including elastic modulus **(c.1** and **d.1),** maximum strain **(c.2 and d.2)** and ultimate tensile strength **(c.3** and **d.3)**. Results are mean ± SEM (n=7). **(e)** Average surface roughness of PCL (10 mg/mL) and PDMS (1/10) nanofilms as evaluated by AFM analyses. Results are mean ± SEM (n=9). **(f)** AFM images of PCL (10 mg/mL) and PDMS (1/10; tapping mode; height imaging). Scan areas of 50×50 μm^2 were used. **(g)** Mechanical properties of PLLA nano films. **(g.1)**Elastic modulus .Results are mean± SEM (n=4); (**g.2)** Average height and **(g.3)** Average roughness. Results are mean± SEM (n=9). In **(d)**, **(e)** and **(g.3)** statistical analyses were performed by an ANOVA test followed by a Dunnet´s multiple comparison post-test. In **(b)**, **(c)** and **(g.1)** and **(g.2)** statistical analysis were performed by Kruskall Wallis test followed by a Dunn´s multiple comparison test. *p<0.5, **p<0.1, ***p<0.001, ****p<0.0001.

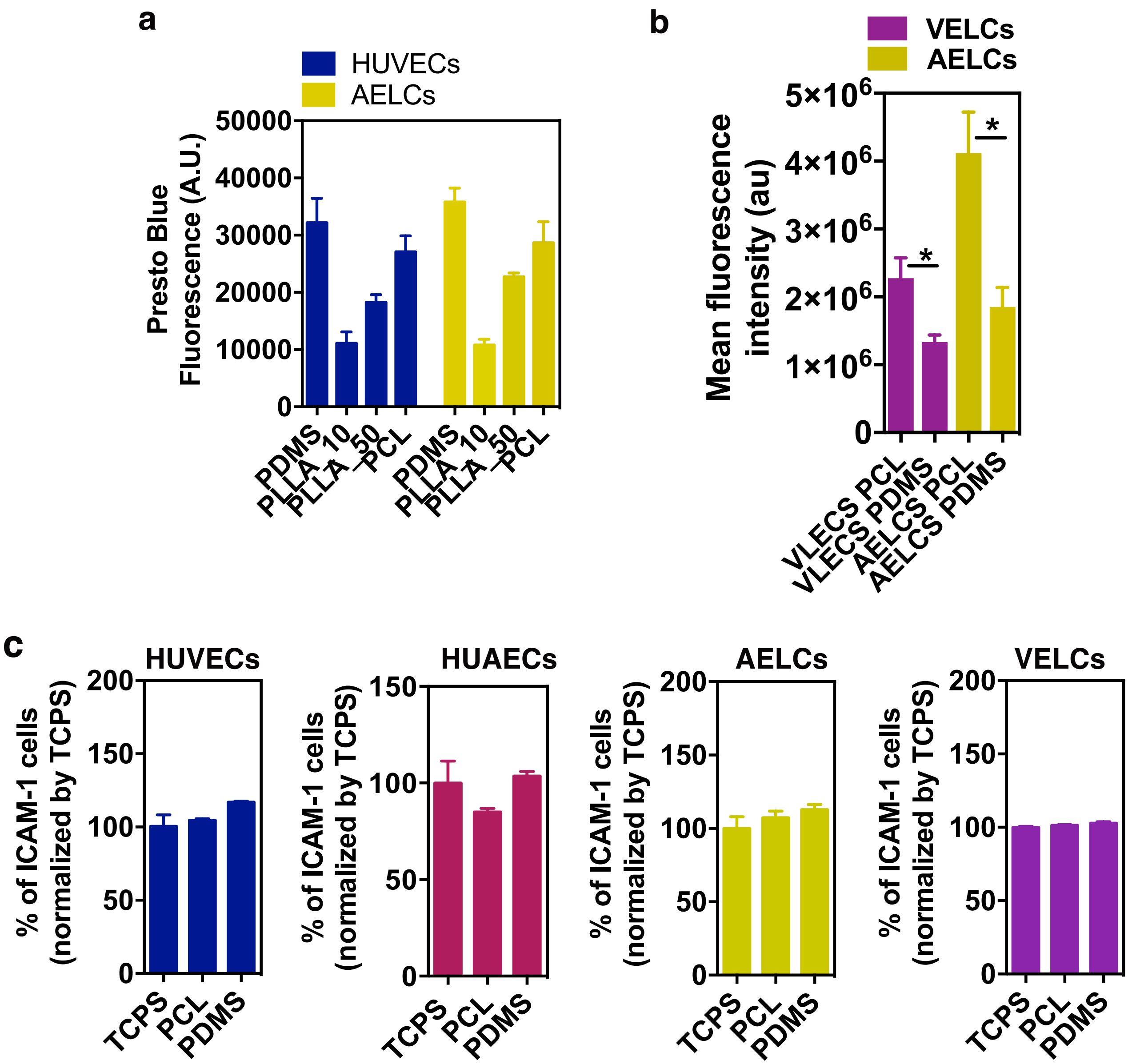

Supplementary Figure S9- Cell viability, monolayer formation and pro-inflammatory response of AELCs and VELCs. (a) Viability of HUVECs and AELCs cultured in PLLA, PCL and PDMS nanofilms for three days, as assessed by a Presto Blue assay. Results are average ± SEM (n=3-6). PLLA_10 and PLLA_50 means PLLA nanofilms prepared from 10 or 50 mg/ml of PLLA. **(b)** Monolayer formation of VELCs or AELCs cultured in PDMS or PCL nanofilms for 3 days. Mean fluorescence intensity for the immunostainings of VE-Cadherin. Results are mean ± SEM (n=4). Statistical significance was evaluated by a Mann-Whitney test. **(c)** Flow cytometry analyses for the basal expression of ICAM-1 in HUVECs, HUAECs, VELCs and AELCs after 3 days in culture in TCPS, PCL or PDMS nanofilms. Data was normalized as percentage relative to TCPS. Results are mean ± SEM (n=3). Statistical significance was evaluated by Kruskal-Wallis test. *p<0.5.

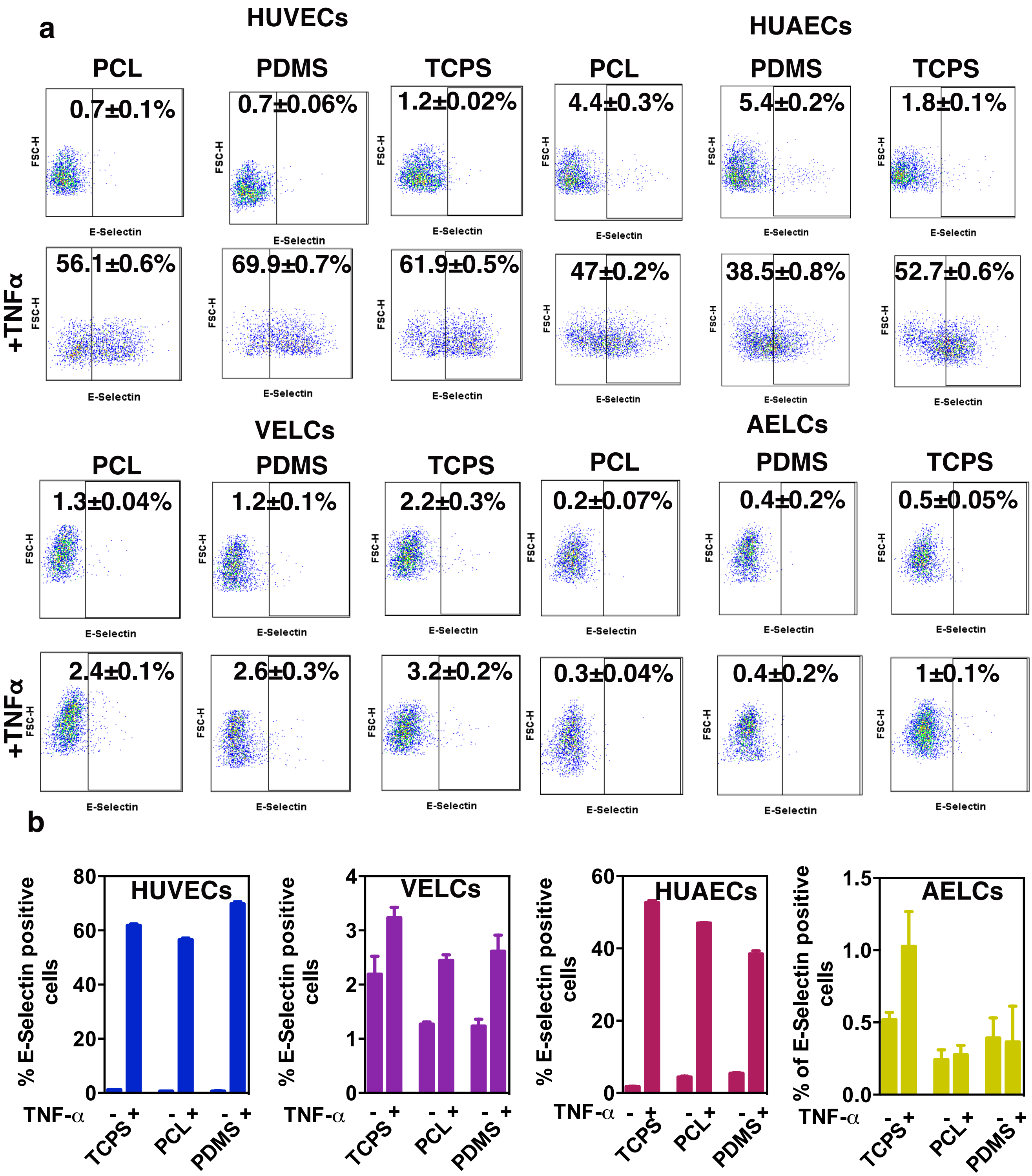

Supplementary Figure S10- Inflammatory profile of the different cell types on nanofilms. **(a)** Representative flow cytometry scatter plots for the expression of E-Selectin in HUVECs, HUAECs, AELCs and VELCs cultured in PCL (10 mg/ml) nanofilms, PDMS (1/10) nanofilms and TCPS for 72 h before and after exposure to TNFα (10 ng/ml) for 24h. Results are average ± SEM (n=3). Shift of the protein of interest was calculated based on the isotype controls which had 1% overlap. **(b)** Plots with the average expression before and after exposure TNFα (10 ng/ml) for 24h. Results are average ± SEM (n=3).
